# Phage cocktails: state-of-the-art technologies and strategies for effective design

**DOI:** 10.1093/femsre/fuaf061

**Published:** 2025-12-08

**Authors:** Tomoyoshi Kaneko, Kanata Nakatsuka, Satoshi Tsuneda

**Affiliations:** Department of Life Science and Medical Bioscience, Waseda University, Tokyo, 162-8480, Japan; Phage Therapy Institute, Comprehensive Research Organization, Waseda University, Tokyo, 162-8480, Japan; Department of Life Science and Medical Bioscience, Waseda University, Tokyo, 162-8480, Japan; Phage Therapy Institute, Comprehensive Research Organization, Waseda University, Tokyo, 162-8480, Japan; Department of Life Science and Medical Bioscience, Waseda University, Tokyo, 162-8480, Japan; Phage Therapy Institute, Comprehensive Research Organization, Waseda University, Tokyo, 162-8480, Japan

**Keywords:** phage therapy, phage cocktail, phage resistance, ESKAPE, AMR, bacteriophage, clinical application

## Abstract

With the growing severity of antimicrobial resistance (AMR), phage therapy has garnered attention as a novel therapeutic alternative. In particular, phage cocktails, which combine multiple phages, potentially offer broader antimicrobial spectra than single-phage applications and may suppress the emergence of resistant bacteria. This comprehensive review systematically examines cutting-edge technologies and effective strategies for designing phage cocktails. Special attention was given to the combination of phages recognizing different receptors, designs based on phage-bacteria infection network analysis, and synergistic effects with antibiotics. Additionally, the analysis of large-scale clinical studies has identified challenges in practical implementation, including ensuring cocktail stability and addressing immune responses. These insights are expected to contribute to the design of more effective phage cocktails and the establishment of novel therapeutic strategies to address AMR.

## Teaser

This comprehensive review systematically examines cutting-edge design principles for phage cocktails, focusing on strategies to prevent antimicrobial resistance through receptor diversity, phage–antibiotic combinations, and network-based approaches.

## Introduction

In recent years, the antimicrobial resistance (AMR) crisis has continued to escalate, necessitating the urgent development of novel infectious disease treatment modalities (Papanicolaou et al. 1990, Horii et al. 1993, Doi et al. 2002, Mirzaei and Nilsson 2015). The World Health Organization recognizes AMR as a critical challenge and promotes countermeasures (Lawe Davies 2017). In particular, a group of pathogenic bacteria collectively known as ESKAPE (*Enterococcus faecium, Staphylococcus aureus, Klebsiella pneumoniae, Acinetobacter baumannii, Pseudomonas aeruginosa*, and *Enterobacter* species) has acquired substantial multidrug resistance, presenting serious problems in clinical settings (Rice 2008). Additionally, *Escherichia coli*, although not part of the original ESKAPE classification, represents another critical priority pathogen owing to its widespread antibiotic resistance and clinical relevance. A comprehensive analysis published in 2024 estimated that AMR was associated with 4.71 million deaths in 2021, with 1.14 million directly attributable to AMR, and estimated that annual deaths attributable to AMR will increase to 1.91 million by 2050 (Naghavi et al. 2024). Furthermore, the authors predicted that the cumulative number of deaths attributable to AMR between 2025 and 2050 would approach 39.1 million (Naghavi et al. 2024).

Under these circumstances, infectious disease treatments utilizing bacteriophages (phages), which are viruses that infect bacteria, have garnered attention. As recently outlined by Skurnik et al., phage therapy was temporarily eclipsed by the widespread adoption of antibiotics. Nevertheless, with the intensification of the AMR crisis and advancements in modern technology, the value of phage therapy, which offers specific antibacterial action with minimal side effects, is being re-evaluated on a more scientific foundation (Skurnik et al. 2025).

In phage therapy, the use of phage cocktails (combinations of multiple phages) furnishes numerous advantages over single-phage applications. First, a broader antimicrobial spectrum can be achieved (Chen et al. 2024). Given that several phages exhibit extremely narrow host specificity (Nobrega et al. 2018), combining multiple phages enables efficacy against a wide range of strains (Santamaría et al. 2014, Mapes et al. 2016). Second, phage cocktails can suppress the emergence of phage-resistant bacteria. A well-known approach involves combining phages that recognize distinct receptors, thereby reducing the probability of bacteria simultaneously acquiring resistance against all phages (Tanji et al. 2004).

However, multiple factors need to be considered to ensure the design of effective phage cocktails. Although phages are generally considered safe, certain phages may stimulate the host immune system and induce inflammatory responses (Bellegh et al. 2017, Gogokhia et al. 2019, Sweere et al. 2019). In addition, antibody production against phages may affect their therapeutic efficacy, highlighting the importance of countermeasures (Berkson et al. 2024). Furthermore, the characterization of individual phages comprising the cocktail, phage–phage interactions, and optimal combination numbers remains unexplored. Technical challenges for practical implementation, such as cocktail stability and quality control, also exist. Therefore, establishing standardized indicators to evaluate phage cocktails is desirable.

Studies on phage cocktails targeting various pathogenic bacteria have been reported. The development of phage cocktails against the ESKAPE bacterial group is particularly active, with their efficacy verified in diverse experimental systems, ranging from *in vitro* to *in vivo*. These studies proposed various approaches regarding the selection criteria for phages constituting cocktails and their combination methodologies.

This review systematically organizes the current knowledge regarding phage cocktail design and comprehensively summarizes the criteria for their formulation. Particular focus was placed on the selection criteria for constituent phages, along with a detailed examination of the characteristics, advantages, and challenges of each methodology. Technical challenges for practical implementation and prospects are also discussed. This review aimed to contribute to the advancement of phage therapy by providing insights into effective phage cocktail designs.

## Phage acquisition and characterization

### Isolation methods from environmental samples

#### Selection of isolation sources and their importance

The first step in phage cocktail preparation is to obtain the appropriate phages. Although phages exist ubiquitously in nature, including in extreme environments and human habitats, selecting suitable sources is essential for efficient isolation (Mattila et al. 2015, Musheigan 2020). A crucial point in source selection is choosing environments where the target pathogenic bacteria exist or are likely to exist; from an evolutionary perspective, such environments are more likely to contain phages with high lytic activity against the target bacteria. Phage isolation from multiple environments increases the likelihood of obtaining diverse phages (Maffei et al. 2021). Even within the same environment, phage diversity may fluctuate seasonally (Tanji et al. 2003). For cocktails that combine multiple phages, careful isolation and source selection are necessary to ensure diverse phage characteristics. As a case in point, hospital and medical sewage wastewater represent highly effective sources, as phages isolated from these environments demonstrate superior lytic activity against clinical isolates, with one study showing that medical sewage yields phages capable of infecting up to 96.3% of carbapenem-resistant *A. baumannii*(Li et al. 2020). Animal waste disposal sites, including cattle and buffalo farm waste, as well as dairy farm effluents, serve as rich sources of diverse phages with broad host ranges against enteropathogenic bacteria (Shende et al. 2017). Urban sewage systems consistently provide phages with rapid lytic activity, while hospital-derived samples offer phages specifically adapted to clinically relevant resistant strains (Yamamura et al. 2022, Sada and Tessema 2024). For foodborne pathogens, fresh food samples, particularly seafood, can serve as direct sources of pathogen-specific phages, although success depends on the presence of target bacteria in relatively high concentrations (Tan et al. 2021).

#### Screening methods

In phage screening, the selection of host bacteria directly affects phage diversity and therapeutic efficacy. There are both advantages and disadvantages in selecting either clinical isolates or laboratory strains as host bacteria for screening. The use of clinical isolates potentially yields more diverse phages because each strain has a distinct receptor structure and defence mechanism (Gaborieau et al. 2023, Costa et al. 2025). Clinical isolates also enhance the likelihood of obtaining phages relevant to actual infections. Phages isolated from clinical isolates demonstrate taxonomic diversity and high lytic activity against similar clinical isolates (Finney et al. 2022). Instead of human-derived clinical isolates, animal-derived isolates such as Avian-Pathogenic *E. coli* or colitis mouse faecal *E. coli* have yielded phages belonging to novel lineages that remain effective against human-derived clinical isolates, supporting the value of utilizing diverse strains as hosts for phage acquisition (Nicolas et al. 2023, Kaneko et al. 2026). However, safety concerns restrict the application of these clinical isolates in some laboratory facilities.

By contrast, laboratory strains are easy to handle, reproducible, and universally available across research institutions worldwide. Although the use of only laboratory strains does not encompass the diversity of clinically problematic pathogens, thereby potentially limiting clinical efficacy, studies have reported that employing multiple screening sources can maintain phage diversity even with laboratory strains, with these phages demonstrating partial effectiveness against clinical isolates (Maffei et al. 2021, Abigail et al. 2023, Humolli et al. 2025, Kaneko et al. 2025a).

Ideally, diverse host strains (including standard laboratory strains and local clinical isolates) should be used during the initial screening stage. In particular, the use of multiple host strains with different genetic backgrounds and phenotypic characteristics (e.g. drug resistance patterns, biofilm formation capabilities, and capsule types) facilitates the efficient isolation of various phages. When facility conditions are limited, focusing on laboratory strains while utilizing as many diverse screening sources as possible enables the acquisition of phages with broad characteristics.

Appropriate concentration methods and isolation methods are crucial for efficient phage isolation. Polyethylene glycol (PEG) precipitation is widely used to concentrate phages from environmental samples (Tanji et al. 2005, Nicolas et al. 2023). This method is particularly effective for concentrating phages from large volumes of environmental water. Specifically, PEG 6000 (10 w/v %) and NaCl (4 w/v %) are added after removing solids from the environmental samples by centrifugation. Following overnight incubation at 4°C, phages are recovered as precipitates by centrifugation and resuspended in a small volume of buffer. Finally, chloroform treatment is used to remove environmental bacteria. This method enables efficient phage concentration from several litres of environmental samples. However, certain phages may become inactivated during this process. For instance, enveloped phages are susceptible to chloroform (Laurinavičius et al. 2004). Filtration methods are effective for isolating such phages (Zucker et al. 2022). Furthermore, filtration methods are effective in isolating jumbo phages, which are expected to possess various functions (Hu et al. 2023).

The double-layer agar method can effectively isolate phages from screening sources (Maffei et al. 2021, Nicolas et al. 2023). This standard method for reliably isolating single-phage clones comprises the following steps. Initially, soft agar (~0.5%) containing phage samples and host bacteria is overlaid on the lower agar medium. After overnight incubation, the desired plaques are selected based on characteristics, such as size, transparency, and shape, and the isolation procedures are repeated 2–3 times to obtain pure phage clones.

Liquid culture isolation methods are alternatives to conventional plate culture methods (Jiang et al. 2020, Quirós et al. 2023). These methods are particularly effective for bacteria that form colonies poorly on agar media (e.g. nitrifying bacteria) or bacteria where excessively dense growth makes individual plaque formation difficult. Phages can be detected by measuring temporal changes in culture turbidity.

Recently, droplet-based methods have been reported as efficient screening techniques (Hoshino et al. 2023). Water-in-oil droplets are utilized as reaction chambers for co-culturing phages and host bacteria within individual droplets. This approach is expected to achieve a higher throughput than conventional methods and is particularly effective for screening phages targeting bacteria that are difficult to grow on agar media. Combined with fluorescent staining, the simultaneous counting and isolation of viable phage particles is possible.

### Utilization of phage banks

Phage banks are important resources that provide quality-assured and diverse phages. They are particularly useful when direct phage isolation from the environment is difficult or when phages with known characteristics are required.

Currently, multiple major phage banks exist worldwide. The Félix d’Hérelle Reference Center for Bacterial Viruses (Laval University, Canada) is one of the largest collections of >4000 phage strains globally. It contains abundant phages against pathogenic bacteria, enabling further research. The Eliava Institute in Tbilisi, Georgia (founded by George Eliava in 1923, with Félix d’Hérelle playing a pivotal role in its establishment), emphasizes the collection and preservation of phages, specifically for clinical applications, supported by Eastern Europe’s longstanding track record in phage therapy. DSMZ (Deutsche Sammlung von Mikroorganismen und Zellkulturen) in Germany provides diverse phage collections alongside quality-controlled various microbial strains. The American Type Culture Collection (ATCC) in the United States also offers research phages under a standardized quality control system, including verification of phage identity, purity, and viability through standardized protocols.

One advantage of these phage banks is the availability of quality-controlled standard strains. Although not applicable to all phages, basic information such as host range and growth characteristics for each phage has been determined, facilitating appropriate phage selection based on specific research or therapeutic goals. Additionally, genomic information is available in some cases.

Nevertheless, phage banks have certain constraints. First, the preserved strains are isolated and maintained against specific standard hosts, necessitating separate testing of their activity against target clinical isolates. Second, the possibility of titre reduction or characteristic changes owing to long-term preservation should be considered. Therefore, post-receipt quality confirmation and titre recovery are recommended when necessary.

Additionally, practical considerations, such as ensuring appropriate transport conditions and export/import procedures based on the regulations of individual countries, are necessary to obtain phages from phage banks. More stringent quality standards and procedures may be required, particularly for clinical applications. Considering these points, the appropriate use of either direct isolation from the environment or acquisition from phage banks, according to the objectives, is expected to lead to effective phage cocktail development.

### Purification methods

The phage on tap protocol is an efficient phage purification method (Bonilla et al. 2016). The basic flow of this protocol involves the stepwise purification of high-titre phage solutions. First, concentrated phage solutions undergo ultrafiltration to efficiently remove impurities such as endotoxins. For more advanced endotoxin removal, several strategies are available, including organic solvent extraction using octanol, where 0.4 volumes of octanol are added to the phage solution to transfer endotoxins to the octanol layer, with efficient endotoxin removal achieved by recovering the aqueous layer (Cumming and Rücker 2017). Commercially available affinity columns, such as EndoTrap HD, provide highly effective endotoxin removal when combined with ultrafiltration, reducing endotoxin-to-phage ratios from 3.5 × 10^4^ EU/10^9^ PFU (plaque-forming units) to 0.09 EU/10^9^ PFU (Hietala et al. 2019). Alternative approaches include anion exchange chromatography combined with enzymatic treatment using alkaline phosphatase.

Various purification methods are available depending on the specific purpose. One representative method is caesium chloride (CsCl) density gradient centrifugation (Nasukawa et al. 2017). This technique involves layering different concentrations of CsCl solution and adding a phage solution to the top, followed by ultracentrifugation. The phage particles form bands at positions corresponding to their densities, enabling the acquisition of highly pure phage suspensions. Although particularly suitable for applications requiring highly purified phages, such as structural analysis, this method is time-consuming and requires expensive ultracentrifugation. An additional complication that prolongs the ultracentrifugation method is the need for dialysis to remove residual cesium chloride from the final phage preparation. The previously mentioned PEG precipitation method has long been used for purification purposes. Despite its relative simplicity and no need for special equipment, complete PEG removal may be difficult in some cases. In addition, because PEG precipitation is a non-specific method applicable to various biological macromolecules including proteins, extracellular vesicles, and collagens (Robern and Gleeson 1978, Ramshaw et al. 1984, Pons Royo et al. 2022, Otani et al. 2025), the precipitate, along with phages, may contain impurities such as host-derived proteins, nucleic acids, and endotoxins, which can interfere with subsequent characterization and application (Shi and Tarabara 2018).

Ion-exchange chromatography can also be used for phage purification (Vandenheuvel et al. 2018). This method is based on the differences in the surface charges of phage particles. Although found to be particularly effective for endotoxin removal, this method requires optimization of conditions such as column chemistry, pH, and ionic strength for each phage (Adriaenssens et al. 2012). Its application may be particularly challenging for phages with low net surface charge, which exhibit weak binding to ion-exchange resins (Brorson et al. 2008).

Each of these purification methods has distinctive features, necessitating selection based on purpose and target phage properties. Specifically, CsCl density gradient centrifugation is most appropriate for applications requiring the highest purity and complete endotoxin removal (up to 99% efficacy), making it ideal for structural studies and clinical applications despite its time-consuming nature. EndoTrap HD affinity columns combined with ultrafiltration are recommended when rapid processing and moderate purification are required, achieving significant endotoxin reduction with shorter processing times (Hietala et al. 2019). Organic solvent extraction methods using octanol or butanol are suitable for cost-effective endotoxin removal when achieving levels below 10 EU/ml is acceptable (Szermer-Olearnik and Boratyński 2015), particularly for large-scale preparations. PEG precipitation followed by chloroform treatment remains appropriate for initial concentration steps when chloroform compatibility is not a concern, although it provides limited purification. Ion-exchange chromatography has shown improved performance when combined with enzymatic pre-treatment and is particularly suitable for removing protein contaminants alongside moderate endotoxin reduction (Van Belleghem et al. 2017). In some cases, a combination of multiple methods may enable a more effective purification, with the optimal strategy depending on the intended application’s purity requirements, processing scale, and time constraints.

### Standardization of characterization methods

Accurate evaluation of constituent phage characteristics is essential for effective phage cocktail design. However, evaluation methods frequently differ between reported studies, complicating the comparison and reproduction of results. Therefore, it is necessary to establish standardized evaluation indicators.

#### Host range evaluation

One of the most fundamental characteristic evaluations is the determination of the host range. This is particularly critical because phages typically exhibit extremely narrow host specificities, with each phage exhibiting a unique infection pattern against distinct bacterial strains. Therefore, the host range profile is one of the most distinctive and characteristic features of a phage and essentially provides a ‘fingerprint’ of its biological activity. The direct spot test (DST) or efficiency of plating (EOP) is employed to determine the host range (Mirzaei and Nilsson 2015, Fong et al. 2021, Haines et al. 2021). DST is widely used and is considered the most straightforward host range evaluation method (Mirzaei and Nilsson 2015). This method involves directly dropping an undiluted or minimally diluted phage solution onto host bacterial lawns and assessing the lysis pattern at the spot area. The results are categorized into three types: complete lysis (clear zone), partial lysis (turbid zone), and no lysis. This technique offers the advantage of rapid screening for infectivity against numerous host strains. However, false positives can occur through several mechanisms (Mirzaei and Nilsson 2015). Phages may adsorb onto bacteria they cannot productively infect and cause cell lysis through mechanical disruption (‘Lysis from Without’) or abortive infection (Abedon 2011). Additionally, phage-encoded lytic enzymes such as endolysins or spanins present in the phage lysate can cause bacterial lysis independent of infection. Bacterial-derived bacteriocins contaminating the phage preparation may also contribute to clearing zones (Mirzaei and Nilsson 2015). Furthermore, the presence of phage defence systems in host bacteria can result in apparent clearing without productive infection, contributing to overestimation of host range in spot test results (Mirzaei and Nilsson 2015). These mechanisms may produce apparent clearing without productive infection or sufficient progeny phage formation, leading to overestimation of host range in spot test results (Kutter 2009). The EOP is used to prevent host range overestimation and enable a more quantitative evaluation. This is calculated by dividing the number of plaques formed on target host strains by the number formed on standard host strains. By quantifying actual plaque formation rather than relying on visual clearing zones, EOP assays can differentiate true productive infections from non-productive interactions. For instance, restriction-modification systems may permit visible clearing in spot tests but result in plaque counts several orders of magnitude lower in EOP measurements (Mirzaei and Nilsson 2015). However, results may vary even with identical phages depending on culture conditions and host physiological states, necessitating the detailed recording of experimental conditions. Several factors significantly influence host range evaluation outcomes. Bacterial growth phase is critical, as exponential-phase cultures typically demonstrate higher susceptibility to phage infection compared with stationary-phase cultures due to differences in metabolic activity and cell surface receptor expression (Kutter 2009). Culture medium composition affects both bacterial physiology and phage adsorption efficiency, with nutrient-rich media generally supporting better phage propagation, while specific medium components can influence cell surface properties and receptor availability. Temperature variations can alter both bacterial growth rates and phage infectivity, with optimal temperatures typically ranging from 35°C to 37°C for most human pathogenic bacteria, although deviations can significantly impact infection efficiency. The age of bacterial cultures influences cell wall integrity and receptor expression patterns, with fresh overnight cultures often providing more consistent results than aged cultures. Additionally, the presence of divalent cations such as Ca^2+^ and Mg^2+^ in the culture medium can be essential for phage adsorption and injection in many phage-bacteria systems (Chhibber et al. 2014). pH variations can affect both bacterial cell surface charge and phage stability, potentially leading to false negative results if conditions deviate significantly from optimal ranges (typically pH 6.5–8.0). These physiological variables can lead to variations of up to several orders of magnitude in apparent host range results, emphasizing the importance of standardizing culture conditions, using consistent bacterial growth phases, and maintaining detailed experimental protocols for reproducible host range determination.

#### Lytic activity evaluation

Multiple quantitative approaches are available for evaluating phage lytic activity, each providing distinct insights into different aspects of phage–bacteria interactions. The Virulence Index (VI) quantifies lytic activity by comparing the growth curves of phage-infected and uninfected control groups (Storms et al. 2020). This approach involves infection experiments at different multiplicities of infection (MOIs) using 96-well plates with temporal turbidity measurements, calculating the area under the curve for infected (Ai) and control (A0) groups to determine local lytic activity (VI = 1–Ai/A0). Similarly, PhageScore provides standardized approaches for quantifying phage lytic activity based on bacterial reduction curves, with studies showing high correlation (r = 0.94) between VI and PhageScore, indicating they capture similar aspects of phage–bacteria interactions during the acute phase of infection (Konopacki et al. 2020, Sørensen et al. 2021). The Suppression Index evaluates bacterial growth suppression over fixed time periods (30 hours in the study), providing insights into longer-term phage effectiveness beyond immediate lytic effects (Kim et al. 2024). These existing metrics primarily focus on immediate bactericidal effects and early phases of phage–bacteria interaction, which may have limitations in predicting long-term therapeutic success when bacterial regrowth occurs following initial suppression. The advantage of these quantitative methods lies in their ability to provide objective comparisons of phage lytic activity under different conditions and to enable systematic cocktail design. However, the ability to suppress the emergence of resistant bacteria has not been evaluated using these approaches, highlighting the need to develop new evaluation methods that capture the temporal dynamics of sustained bacterial suppression.

#### Whole-genome analysis

Phage genome analysis is essential for safety assessment and characterization of phages used in cocktails. In particular, analysing the gene regions involved in host recognition (e.g. tail fibres) provides crucial information for designing combinations of phages that recognize distinct receptors. Pharokka, a phage genome-specific tool, is recommended for the identification and annotation of a given phage gene (Bouras et al. 2023). Online accessible RAST and DFAST are also valuable, user-friendly tools (Aziz et al. 2008, Tanizawa et al. 2016, 2018). Although RAST provides detailed functional annotations, it requires a longer processing time, whereas DFAST enables rapid analysis. In addition to host recognition gene regions, exploring sequences related to safety such as lysogenic factors (e.g. integrases for genome integration, repressors for maintaining lysogeny, and excisionases for prophage excision), toxin genes, and antibiotic resistance genes is essential (Waldor and Friedman 2005). These analyses enable not only the safety confirmation of phages used in cocktails, but also the design of effective combinations based on genetic diversity.

Information regarding phage lineages can be useful in phage cocktail formulations. This systematic classification can be determined using tools such as vConTACT2 on whole-genome data to construct gene-sharing networks between viral genomes and estimate closely related species (Bin Jang et al. 2019). Additionally, PhageClouds enables rapid similarity searches against a database of ~640 000 phage genomes and is valuable for finding closely related species (Rangel-Pineros et al. 2021).

#### Quality and safety evaluation

##### Genetic-level safety evaluation

For the clinical application of phage cocktails, confirming the absence of toxin genes, antibiotic resistance genes, and genes associated with lysogeny is crucial for safety evaluation. In particular, toxin and antibiotic resistance genes require careful evaluation during screening because of their potential for transduction into pathogenic bacteria (Waldor and Friedman 2005). Accordingly, specialized databases and tools, such as VirulenceFinder and ResFinder, are available to undertake this evaluation (Kleinheinz et al. 2014, Florensa et al. 2022).

An additional critical safety consideration is the potential contamination of therapeutic phage preparations with prophages induced from lysogenic host strains used for phage propagation (Rotman et al. 2012, Alexander et al. 2017, Pei et al. 2021). Such contaminating temperate phages pose serious risks, as they frequently encode virulence determinants or antibiotic resistance genes characteristic of pathogenic bacteria (Zhu and Mathur 2022, Gu et al. 2023). This contamination can lead to misinterpretation of host range specificity and potentially cause undesirable therapeutic outcomes. Therefore, careful selection of non-lysogenic host strains for phage propagation and rigorous screening for temperate phage contamination in final preparations are essential safety measures. PHASTER analysis can help detect prophage sequences (Arndt et al. 2016) and whole-genome sequencing of both phages and host bacteria should be performed to identify phage sequences related to lysogeny, such as integrases and repressors, as well as genes encoding toxins or antibiotic resistance.

##### Immune response evaluation

Although phages are generally considered safe for eukaryotic cells because of fundamental structural differences between prokaryotes and eukaryotes, several potential risks must be considered (Cui et al. 2024). Some phages stimulate the host immune system and induce inflammatory responses (Gogokhia et al. 2019). Phage DNA can be recognized by pattern recognition receptors such as TLR9 and TLR3, triggering type I interferon production (Gogokhia et al. 2019, Sweere et al. 2019, Podlacha et al. 2024). This immune response has multiple biological consequences: it may induce inflammatory cytokine production, potentially exacerbating conditions such as colitis in susceptible individuals (Gogokhia et al. 2019, Sweere et al. 2019); it can trigger antiviral immunity that suppresses antibacterial responses, thereby impairing bacterial clearance and potentially reducing therapeutic efficacy (Sweere et al. 2019, Podlacha et al. 2024); and it may also promote anti-inflammatory cytokine production, which could contribute to the safety profile of phage therapy (Sweere et al. 2019). In addition, the production of phage-specific neutralizing antibodies can reduce therapeutic efficacy and warrants the development of effective countermeasures (Berkson et al. 2024). Furthermore, toxicity due to impurities such as endotoxins in phage preparations must be considered. The maximal level of endotoxins for intravenous applications of pharmaceutical and biological products is set at 5 endotoxin units (EU), that is, 500 pg of endotoxins per kg of body weight per hour (Daneshian et al. 2006). For other administration routes, the acceptable limits may vary, with topical applications generally allowing higher endotoxin levels than systemic administration. Effective endotoxin removal can reduce contamination from initial levels of 10^3^–10^5^ EU/ml in crude lysates to clinically acceptable levels below 5 EU/ml through appropriate purification strategies (Van Belleghem et al. 2017). These safety evaluations are important for the clinical application of phage cocktails.

#### Stability evaluation

The evaluation of phage stability is another crucial element (Duyvejonck et al. 2021). Stability against environmental factors such as temperature, pH, and ionic strength is directly related to storage conditions and effectiveness in various application scenarios (Bagińska et al. 2024, Pirnay et al. 2024). For assessing temperature effects, storage at 4°C, -20°C, -80°C, and in liquid nitrogen is common. It is particularly important to determine the long-term storage stability with protective agents such as glycerol, dimethyl sulfoxide, or sucrose (Duyvejonck et al. 2021). Titre reduction due to repeated freeze-thaw cycles must also be considered. Additionally, phage stability should be evaluated under various pH conditions and ionic strengths relevant to intended applications. Light exposure and oxidative stress during storage can also affect phage viability, necessitating evaluation of storage in opaque containers and under controlled atmospheric conditions when appropriate (O’Connell et al. 2022, Malik et al. 2023). These evaluations are crucial for identifying conditions necessary to maintain the structural stability and infectivity of the phages. The interaction with serum is a particularly important evaluation item for clinical applications (Mutti et al. 2023). Physical stress from vibration and agitation during handling and transportation can compromise phage stability. As biological entities, phages may be susceptible to mechanical stresses such as shearing forces encountered during processing and delivery (Flint et al. 2023). The evaluation of mechanical stability and implementation of protective measures, such as optimized buffer compositions or cushioning materials, should be considered when necessary.

The evaluation of storage stability is especially important when using phage cocktails. Individual phages within cocktails may be stable alone but could experience titre reduction after mixing due to interactions such as phage aggregation. For instance, the PhagoBurn trial reported a substantial reduction in the titre after cocktail preparation (Jault et al. 2019). Therefore, it is crucial to evaluate the stability of both individual phages and post-cocktail preparations.

The evaluation results provide critical data for optimizing storage methods and determining formulation strategies. However, the evaluation conditions and methods must be established by considering the intended use environments. More stringent stability evaluations are required for clinical applications.

## Design principles of phage cocktails

Beyond individual phage characterization, various factors must be considered when combining multiple phages for a cocktail design. This section comprehensively examines the elements necessary for an effective cocktail design.

### Benefits of phage cocktail formulation

For clinical applications, there are two possible concepts for phage cocktailing: ‘prêt-à-porter’ (ready-to-wear) and ‘sur-mesure' (custom-made) (Chan et al. 2013, Haines et al. 2021, Abedon et al. 2021, Skurnik et al. 2025). The prêt-à-porter approach utilizes pre-prepared fixed cocktails. To ensure that phages are effective against specific bacteria, phages with different infection spectra are mixed to lyse various bacterial strains. This approach enables rapid administration, reduces manufacturing costs, and facilitates relatively straightforward quality control (Suleman et al. 2025). However, as demonstrated in the PhagoBurn and ELIMINATE trials, cocktail stability and efficacy prediction may be challenging (Jault et al. 2019, Kim et al. 2024). In particular, the effectiveness against infectious bacteria in individual patients remains uncertain, and the risk of resistant bacterial emergence may be higher than that associated with an individualized approach (Suleman et al. 2025).

The sur-mesure (individualized) approach involves selecting phages individually against bacteria isolated from patients and conducting sensitivity tests prior to use. Because bacteria easily develop resistance to phages via mutations, it is recommended to combine phages with distinct infection mechanisms to prevent the development of resistance. When bacteria are challenged with phages with different infection mechanisms, they must adapt to both mechanisms, reducing the probability of resistance development. The sur-mesure approach offers high efficacy through specialization for specific bacterial strains and minimization of the emergence of resistant bacteria (Abedon et al. 2021). For example, in the Patterson case against multidrug-resistant (MDR) *A. baumannii* infection, individually selected effective phages against patient isolates led to successful treatment (Schooley et al. 2017). However, challenges include time requirements for manufacturing and quality assessment, potentially higher manufacturing costs, and difficulties in responding to emergency infections (Suleman et al. 2025).

The choice between approaches depends on infection severity and urgency. Generally, the sur-mesure approach is more suitable for severe or refractory infections, whereas the prêt-à-porter approach may be more appropriate for acute infections (Abedon et al. 2021). Regionally specific pathogen distributions and resistance patterns must also be considered (Suleman et al. 2025). Establishing manufacturing and quality control systems for both approaches is essential for leveraging their respective advantages (Suleman et al. 2025).

### Criteria for selection of constituent phages

Even when cocktails from phages meet the safety requirements explained in Section “Quality and Safety Evaluation”, multiple important evaluation criteria need to be addressed. First, because phages generally exhibit extremely narrow host specificity, a detailed evaluation of the effectiveness of each phage against various strains is crucial (evaluation methods are detailed in “Host Range Evaluation”). Thereafter, it is essential to confirm that selected phages possess sufficient lytic activity. Quantitative indicators such as the VI should be used to evaluate the lytic activity of individual phages and select effective combinations (Storms et al. 2020). In addition to basic characteristics, such as host range, burst size, and latent period, these parameters are essential for predicting phage performance in clinical applications and ensuring consistent therapeutic efficacy. The effects of environmental factors that influence formulation stability, including temperature, pH, and ionic strength, on lytic activity, should be considered when evaluating selected phages (Kaneko et al. 2023, Sada and Tessema 2024, Sawaengwong et al. 2024). These stability-related factors can significantly impact phage viability and infectivity during storage and *in vivo* application, where conditions may differ substantially from optimal laboratory conditions. Although technically challenging, evaluating activity in actual environments (e.g. wound sites with biofilms) is also important as tissue conditions can affect both phage stability and therapeutic effectiveness (Rezk et al. 2022).

Furthermore, when mixing multiple phages, interactions between phages must be carefully evaluated. Particularly, at the design stage, the possibility that the infection of one phage may inhibit that of another or that the stability of each phage may decrease upon mixing needs to be considered. For instance, in a cocktail against *E. coli*, cases where the expected effects were not obtained owing to phage interactions were reported (Molina et al. 2022).

### Phage combination strategies

Various strategies have been developed for combining phages in cocktail formulations, each with distinct principles, advantages, and technical requirements (Table 1). The following sections provide detailed descriptions of these approaches.

**Table 1.** Comparison of phage cocktail design strategies.

Strategy	Principle	Advantages	Disadvantages	Technical requirements	Representative studies	Target pathogens
Receptor-based approach	Combining phages targeting different cell surface receptors	High resistance suppression, clear mechanistic basis, predictable effectiveness	Requires receptor identification, limited to well-studied bacteria	Resistant strain generation, whole-genome sequencing, comparative genomic analysis	Tanji et al. 2004, Bai et al. 2019, Yang et al. 2020	*E. coli* O157:H7, *Salmonella* typhimurium, *P. aeruginosa*
Infection spectrum-based approach	Combining phages with different host ranges and lytic characteristics	Utilizes readily available data, network-based optimization, machine learning applicable	Dataset dependency: requires diverse strain panels, reduced accuracy with narrow-range phages	Host range matrix construction, PBIN analysis, statistical modelling tools	Molina et al. 2021, Díaz-Galián et al. 2022, Keith et al. 2024	*E. coli*, various clinical isolates
Physiological characteristics-based approach	Combining phages with different physiological characteristics	Reduced strain panel dependency, incorporates phage-specific features, compatible with genomic or target receptor base classification	Requires detailed phage characterization, time-intensive evaluation, limited standardization	Evaluation of physiological characteristics, such as burst size and lysis kinetics	Kaneko et al. 2023, Kaneko et al. 2025b	*E. coli*
Phage-resistant strain utilization	Using phages adapted to or isolated against resistant bacteria	Enhanced bacterial suppression, extended effectiveness duration, historical validation (Appelmans Protocol)	Time-consuming isolation/training, higher manufacturing costs	Phage training protocols, resistant strain isolation, co-evolution monitoring	Borin et al. 2021, Yu et al. 2018, Yoo et al. 2024	*E. coli, P. aeruginosa, K. pneumoniae*
Genomic diversity-based approach	Combining phages with substantially different genome sequences	Simple selection criteria, phylogenetic validation, reduced receptor dependency	Possibility of lacking mechanistic understanding, requires genome sequencing, limited optimization potential	Whole-genome sequencing, phylogenetic analysis, sequence comparison tools	Chen et al. 2018, Stephen et al. 2022, Kaneko et al. 2025b	*E. coli, Aeromonas salmonicida, Enterococcus* spp.
PAC	Combining phage cocktails with antibiotics for synergistic effects	Synergistic antimicrobial activity, resistance suppression, antibiotic resensitization potential	Mechanism-dependent interactions, risk of antagonistic effects, complex dosage optimization	Pharmacokinetic analysis, elucidation of mechanism evaluation	Carmen et al. 2020, Kebriaei et al. 2020, Haeruman et al. 2024, Nakamura et al. 2021	*S. aureus, E. coli, A. baumannii, P. aeruginosa*

Abbreviations: PAC, phage-antibiotic combination; PBIN, Phage-Bacteria Infection Network.

Note: Strategies are not mutually exclusive and can be combined for optimal cocktail design. Selection should be based on target pathogen characteristics, available resources, and clinical requirements.

#### Strategies for combining different phages

**Figure 1.**
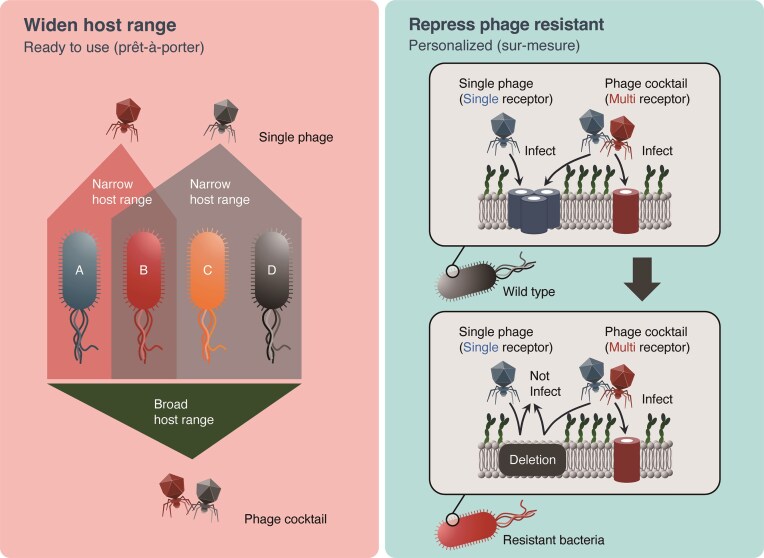
Basic concept of phage cocktail design approaches. Schematic representation of two primary phage cocktail design strategies: prêt-à-porter (ready-to-wear) and sur-mesure (custom-made) approaches. The prêt-à-porter approach emphasizes combining phages with distinct host ranges to achieve broad coverage against diverse bacterial strains, while the sur-mesure approach focuses on designing personalized cocktails that target specific clinical isolates with phages recognizing different receptors to prevent resistance development. The diagram illustrates how these distinct approaches address different clinical requirements.

The prêt-à-porter and sur-mesure approaches described in “Benefits of Phage Cocktail Formulation” have distinct phage cocktail design purposes (Fig. 1). In the prêt-à-porter approach, combining phages that can infect various strains to cover a broad host range is important, with the cocktail’s main purpose being infection spectrum expansion. Conversely, the sur-mesure approach designs cocktails for specific clinical isolates, primarily aimed at suppressing the development of phage resistance. Effective resistance suppression can be achieved by combining phages targeting different receptors (Tanji et al. 2004, Bai et al. 2019, Yang et al. 2020, Chen et al. 2024).

##### Receptor-based approaches

As mentioned in “Strategies for Combining Different Phages”, the most widely recognized phage cocktail strategy against phage resistance development involves combining phages that recognize different cell surface receptors, thereby increasing the fitness costs when pathogens develop phage resistance. For example, combining two phages targeting *E. coli* OmpC outer membrane protein and lipopolysaccharide (LPS) has been shown to markedly delay the emergence of resistant strains (Tanji et al. 2004). Similar approaches have been proven to be effective against *Salmonella enterica, K. pneumoniae*, and *P. aeruginosa* (Bai et al. 2019, Yang et al. 2020, Chen et al. 2024).

**Figure 2.**
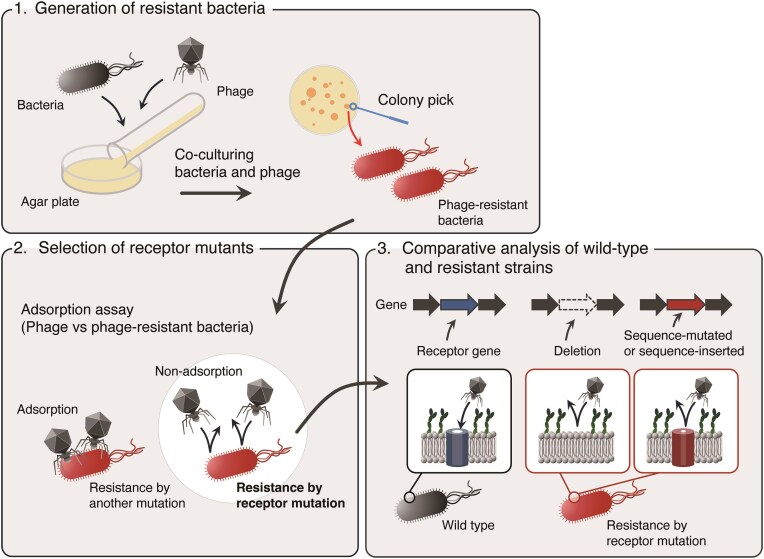
Methodological workflow for phage receptor identification. A diagram illustrating the experimental pipeline for identifying bacterial receptors targeted by phages. The process begins with the isolation of phage-resistant bacterial mutants, followed by whole-genome sequencing to identify the mutated genes. Comparative genomic analysis of wild-type and resistant strains enables the identification of potential receptor genes. The figure highlights the technical challenges of this approach, including the need for both wet-lab expertise in generating resistant mutants and bioinformatics skills for sequence analysis. This receptor identification process is crucial for designing phage cocktails that target different cell surface structures to minimize resistance development.

However, even when phages target the same receptor, the infection mechanisms after receptor matching can differ substantially. This implies that simply selecting random phages from group A (targeting receptor A) and group B (targeting receptor B) based solely on the principle of combining phages with different receptors may not persistently yield optimal results. Post-attachment infection mechanisms, including genome delivery, replication strategies, and lysis, play crucial roles in determining the overall effectiveness of phage cocktails. For instance, if phages targeting different receptors share common vulnerabilities to the same bacterial defence systems (such as restriction-modification systems, CRISPR-Cas systems, or abortive infection mechanisms), both phages may be simultaneously inactivated despite using different entry routes. Similarly, phages with different replication strategies (e.g. lytic vs chronic infection) may exert distinct selective pressures on bacterial populations, affecting the emergence and dynamics of resistance. Therefore, the optimal combination of phages with various infection mechanisms may vary depending on the specific situation and target bacteria. However, the receptor-based approach presents considerable technical challenges. Identifying target receptors warrants the generation of resistant bacteria, followed by whole-genome analysis or comparative genomic analysis with wild-type strains, which requires both time and effort and fundamental knowledge in both dry and wet laboratory techniques (Fig. 2). Furthermore, genomic analysis of phage resistance mutations relies on well-annotated bacterial genome to identify whether mutation genes encode cell surface structures. This approach is challenging for insufficiently characterized bacteria such as *Stenotrophomonas maltophilia*, which are currently acquiring and spreading drug resistance (Gröschel et al. 2020).

##### Combining phages with different infection strategies

The phage infection range provides valuable for information cocktail design without requiring detailed receptor identification. In general, different bacterial strains possess different molecules on their outer membranes/cell walls. Therefore, phage resistance can be suppressed by combining phages that share a common ability to lyse a specific bacterial strain but have distinct host ranges. In addition to focusing on host range differences, attempts have been made to analyse host ranges more systematically, such as phage-bacteria infection networks (PBINs). For instance, a meta-analysis of 35 host range matrices revealed a trade-off relationship between phage host range breadth and lytic exactivity. Considering this trade-off, combining phages with broad host ranges but relatively weak lytic activity with those demonstrating narrow host ranges but strong lytic activity can effectively suppress the emergence of resistant bacteria (Molina et al. 2021). Additionally, by conceptualizing phage-bacterial infection relationships as networks, methods quantifying the importance of each phage (Expected Importance) have been proposed (Díaz-Galián et al. 2022). The importance is calculated from the number of bacteria that a phage can infect (out-degree) and the number of other phages that can infect those bacteria (in-degree). This methodology enables the prediction of combinations that yield maximum lytic effects with minimal phage numbers. Hierarchical clustering of host range matrices allows for efficient selection of phages exhibiting different activity patterns. Furthermore, two complementary PBIN-based design methods have been proposed (Menor-Flores et al. 2022). Although an exhaustive search reliably discovers optimal combinations, the computation time presents challenges. By contrast, the network metrics approach enables practical design timeframes by efficiently narrowing the candidates based on network characteristics.

As a more practical approach, a random forest model using PBIN data successfully predicted phage activity against unknown bacterial strains using interaction data between 314 *E. coli* strains from urinary tract infections and 31 phages (Keith et al. 2024). This model can predict individual phage activity from bacterial genomic features, exhibiting high prediction accuracy (F1 score > 0.6), particularly for phages with broad host ranges (infecting >20% of all strains). Accordingly, this approach enables rapid therapeutic cocktail design based on sequence data obtained from clinical specimens.

These infection range-based methods allow the prediction of effective phage combinations solely through infection spectrum surveys. However, they may result in decreased prediction accuracy if the host bacterial strain diversity used in the infection spectrum surveys is insufficient. Furthermore, the prediction accuracy may decrease when dealing with bacterial strains harbouring phage resistance mechanisms that are not included in the training datasets. Constructing reliable predictive models for phages with narrow host ranges can be challenging, necessitating their integration with experimental screening approaches. Additionally, maintaining the accuracy of the prediction model requires periodic dataset updates and retraining.

Furthermore, combining 13 *E. coli*-infecting phages with different lytic activity characteristics, including lysis time, lysis patterns, and burst size beyond the infection range, was shown to yield more effective therapeutic effects (Kaneko et al. 2023, 2025b). This suggests that the complementary action of phages with different infection strategies effectively suppresses bacterial growth. An attractive aspect of this approach is that it can reduce the dependence on bacterial strain panel diversity for infection spectrum evaluation by incorporating phage-specific features, such as burst size and lysis duration, into the cocktail design.

**Figure 3.**
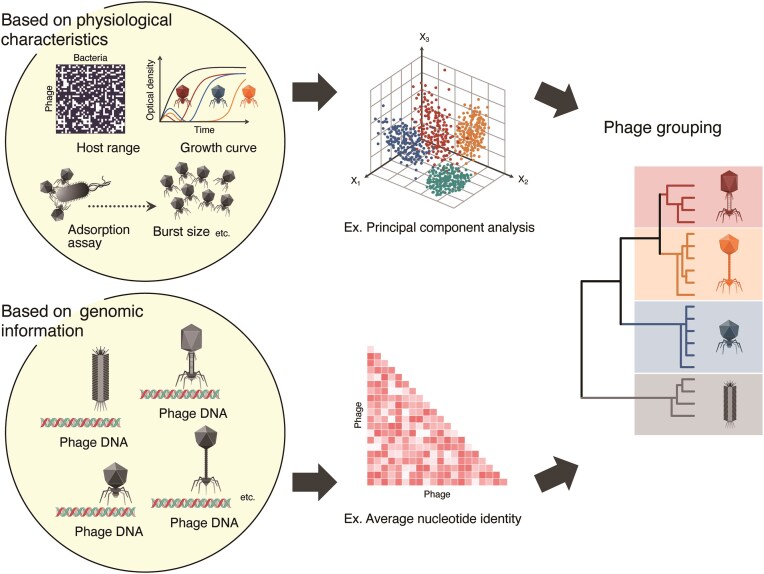
Method of phage grouping for cocktail formulation. Multipanel illustrations revealing different approaches for grouping phages to design effective cocktails. The figure compares two primary methods for determining phage diversity when selecting cocktail components: physiological classification (based on infection characteristics such as burst size, latent period, and lysis patterns) and genomic-based classification approaches (e.g. whole-genome analysis and molecular phylogenetic analysis of tail fibres). These different grouping methods yield similar results in certain cases, suggesting that either approach can be used effectively to ensure that diverse phages are combined into cocktail formulations. Importantly, the diversity of phage types is a key principle for creating effective cocktails that minimize the development of resistance.

Interestingly, classification results based on these physiological characteristics almost completely coincided with whole-genome-based systematic classification, molecular phylogenetic classification based on tail fibres, or classification related to target receptors, suggesting compatibility between these different classification approaches (Fig. 3) (Kaneko et al. 2025b). Genomic differences indicate that differences in phage infection strategies are closely associated with their molecular structures and evolutionary backgrounds. Additionally, the effectiveness of approaches based on differences in infection strategies has been reported.

Five phages against *Aeromonas salmonicida* were isolated, and cocktail effectiveness was verified using various combinations. The results revealed that combining two phages with substantially distinct genome sequences (AS-yj and AS-gz) resulted in markedly higher antibacterial activity than the other combinations (Chen et al. 2018). Similar results have been reported for other for other *Aeromonas* species and *Enterococcus-*infecting phages (Stephen et al. 2022, Fudeshima et al. 2025). These reports suggest the possibility of formulating effective cocktails, primarily based on genomic information, without investigating the target receptors or infection ranges.

##### Design utilizing phage-resistant strains

Design approaches that actively utilize phage-resistant strains have been developed since the early stages of phage therapy. The Appelmans Protocol, developed in the 1920s, is a particularly important methodology (Burrowes et al. 2019). This approach has evolved to include phages adapted to infect phage-resistant bacteria through the continuous co-culture of phages and phage-resistant bacteria. Initially, this method was primarily aimed at expanding the host range. Traditional phage cocktails are regularly updated at the Eliava Institute in Georgia using this method.

**Figure 4.**
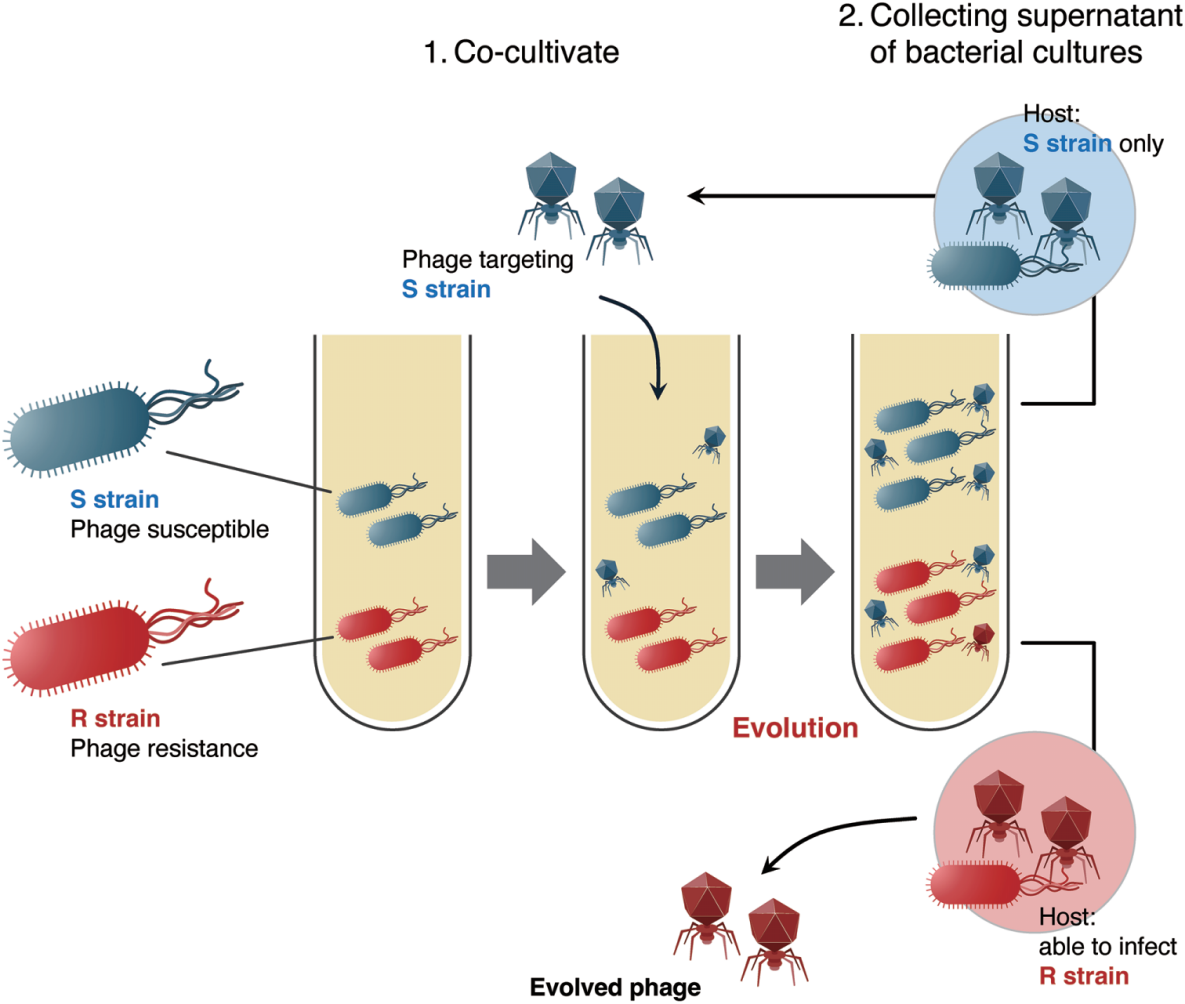
*In vitro* evolutionary strategies for phages utilizing resistant bacterial strains: phage training. Schematic representation of the phage training methodology workflow. This figure illustrates the sequential steps involved in phage adaptation through serial co-culture with phage-resistant bacteria. Initially, the phage is cultured with sensitive and resistant bacteria, followed by phage adaptation through continuous exposure to these resistant strains. Finally, trained phages that can overcome bacterial resistance mechanisms are generated. This methodological approach, based on the principles of directed evolution, allows the development of phages with enhanced infection capabilities against bacterial populations that are resistant to the original phages.

This historical method has evolved into the approach known as ‘phage training’, attracting attention in modern phage therapy development. Recently, approaches for addressing phage-resistant strains have been further expanded, focusing on suppressing the emergence of phage-resistant bacteria and enhancing the efficacy of sustainable phage therapies (Fig. 4). For example, phages trained for ~1 month demonstrated a bacterial suppression capacity ~1000 times stronger than that of untrained ancestral strains, maintaining this effect for 3‒8 times longer (Borin et al. 2021). This enhanced suppression effect stems from multiple factors. Mutations conferring resistance to trained phages were ~100 times less common, indicating that complete resistance required multiple mutations rather than a single step; thus, resistance mutations carried higher fitness costs. Another study demonstrated that host diversity influenced phage adaptation. Reportedly, as the number of host genotypes in a community increases, generalist phages that can infect both the original host and resistant strains are selected over specialist phages that infect only resistant strains, resulting in a slower rate of adaptation to a single host (Sant et al. 2021). These findings suggest that environments containing multiple hosts may yield phages with broader host ranges for designing phage cocktails.

To develop phage cocktails against *P. aeruginosa* strains with different resistance mechanisms, the dsRNA phage phiYY was used to infect *P. aeruginosa* lacking the O-antigen, while the dsDNA phage PaoP5 was trained to develop the adaptive mutant PaoP5-m1, effective against strains with the cleaved O-antigen. Combining these phages with different mechanisms was reported to successfully suppress the emergence of phage-resistant bacteria (Yang et al. 2020).

A broad-host-range phage cocktail against *Pseudomonas aeruginosa* biofilms has been developed using phage-resistant mutants (Mapes et al. 2016). Phage-resistant bacterial strains were induced against initially isolated phages, and novel phages capable of infecting these resistant strains were isolated from environmental samples. The phage cocktail obtained using this method demonstrated a broader host range than single phages and effectively inhibited biofilm formation while reducing existing biofilms. In another study, an *E. coli* ‘guard-killer’ phage cocktail was developed (Yu et al. 2018), with two mutants resistant to phage JDP1 isolated and used to isolate novel phages (RBP and RSP). The combination of JDP1 (which lyses original strains) with RBP and RSP (which lyse resistant strains) resulted in a cocktail that could markedly reduce resistance emergence rates compared with single phages. This ‘guard-killer’ dual-function phage cocktail effectively suppressed resistant bacteria emergence both *in vitro* and *in vivo*.

A recent study adopted a stepwise resistance induction approach to develop phage cocktails against MDR *Klebsiella* (Yoo et al. 2024). In this approach, a new phage was isolated against a bacterial strain resistant to the initial phage, and this process was repeated three times to develop a cocktail of four phages. Notably, these phages could recognize different cell surface molecules, including capsular polysaccharides, FhuA outer membrane proteins, LPS cores, and TonB-dependent transporters. Interestingly, bacteria that acquire sequential resistance demonstrate cumulative trade-off effects, including reduced growth rates, decreased toxicity, and increased biofilm formation. Additionally, ‘re-sensitization’ phenomena were confirmed, where bacteria acquiring resistance to one phage regained sensitivity to another phage.

While these studies have demonstrated the effectiveness of phage cocktail design using phage-resistant mutants, practical challenges are yet to be addressed. In this approach, complementary combinations are created by isolating novel phages capable of infecting resistant strains or by adapting existing phages through training. However, isolating resistant strains, screening for novel phages, or training existing phages is time-consuming, potentially rendering them unsuitable for clinical applications that warrant rapid treatment. Additionally, as the number of phages incorporated into cocktails increases, the manufacturing costs and quality control burdens increase. To overcome these challenges, it is important to accumulate host range data against not only wild-type bacteria but also phage-resistant mutants in phage banks, as well as to establish efficient cocktail design processes for clinical applications.

A recent study has highlighted the potential challenges of the Appelmans Protocol. The application of the Appelmans Protocol to *P. aeruginosa* phage-resistant strains may result in the induction of prophages present in the host bacteria, potentially causing apparent host range expansion (Lee et al. 2024). As discussed in “Genetic-Level Safety Evaluation”, such prophage contamination poses not only the risk of misinterpretation but also serious safety concerns due to the potential transfer of resistance or virulence genes. This could also occur during phage training with similar fundamental principles, emphasizing the importance of genetic verification of input and output phages in phage cocktail experiments to avoid misinterpretation of results and ensure therapeutic safety.

#### Strategies for combining phages and antibiotics

##### Antibiotic combination strategies

**Figure 5.**
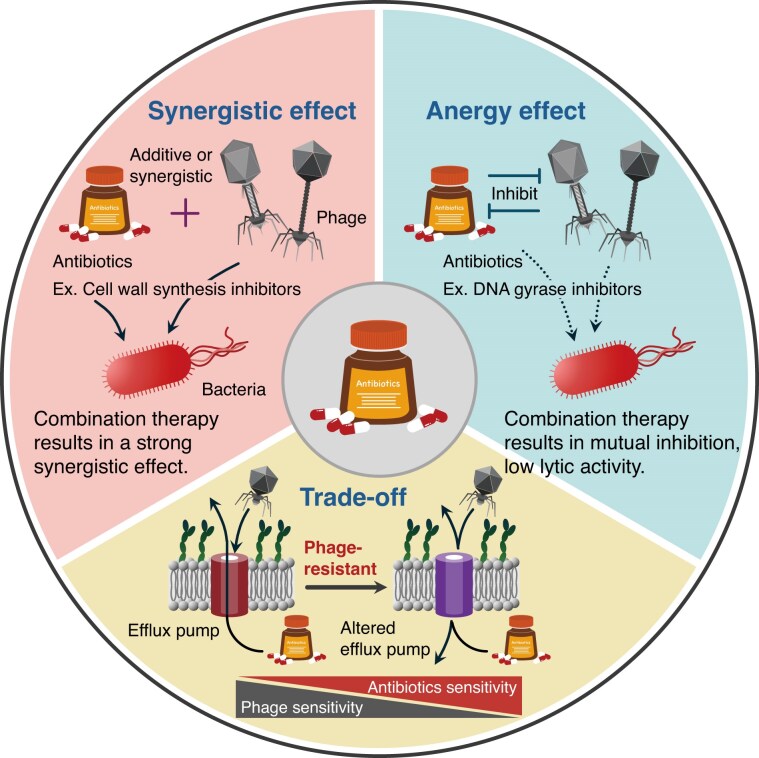
Basic concept of phage and antibiotic cooperation. A schema illustrating key interaction types observed in phage-antibiotic combination (PAC) therapy. Three primary relationships occur when phages and antibiotics are used in conjunction: synergy (enhanced combined effect), anergy (reduced effectiveness in combination), and trade-offs (where resistance to one agent increases susceptibility to the other). The diagram highlights how these distinct interaction patterns influence bacterial responses to combination therapy and demonstrates why understanding these relationships is crucial for designing effective therapeutic combinations. This conceptual framework provides the foundation for the rational selection of phage–antibiotic combinations in clinical applications.

Phage-antibiotic combination (PAC) therapy represents a promising approach with expected synergistic effects and has been extensively investigated (Fig. 5) (Fungo et al. 2023). A systematic analysis of *E. coli* infections has demonstrated that PAC efficacy strongly depends on the mechanism of action of antibiotics (Carmen et al. 2020). In particular, combinations of cell wall synthesis inhibitors (e.g. cefazolin) yield highly synergistic effects, whereas the combination with DNA gyrase inhibitors (e.g. ciprofloxacin) occasionally results in antagonistic effects. Such mechanism-dependent synergistic effects have also been reported in other pathogens. For MDR *A. baumannii*, combining phage pB3074 with cell wall synthesis inhibitors (cefotaxime or meropenem) effectively inhibited and removed biofilms (Luo et al. 2023). Furthermore, the mechanism underlying the synergistic effect was elucidated, whereby phage adsorption and replication are promoted in the presence of antibiotics.

Antagonistic interactions between phages and antibiotics have also been reported. For example, a study using polyvalent phage SaP7 against *Salmonella* and *E. coli* demonstrated antagonistic effects with β-lactam antibiotics, including amoxicillin and cefepime, both *in vitro* and in animal models (Ma et al. 2022). The mechanisms include mutual inhibition, in which antibiotics prevent phage plaque formation, whereas the phage reduces bacterial susceptibility to antibiotics. Additionally, a comprehensive analysis of phage–antibiotic interactions across different antibiotic classes has revealed that certain combinations, such as with chloramphenicol, can result in antagonistic effects, depending on the stoichiometry of the agents (Carmen et al. 2020).

Important advantages of PAC therapy include the suppression of phage-resistant bacterial emergence and the potential restoration of antibiotic effectiveness against existing resistant bacteria (Fig. 5). For example, combining phage Sb-1 with daptomycin against *S. aureus* yielded higher bactericidal effects than standalone use, while suppressing the development of phage resistance (Kebriaei et al. 2020). Additionally, the combination of ciprofloxacin with phage cocktails demonstrated effective bactericidal activity for over 4 days in an *ex vivo* model of *P. aeruginosa* endocarditis (Ghali et al. 2023).

However, clinical application of PAC therapy faces several challenges. First, the effectiveness of PAC therapy is found to be substantially reduced in biological environments such as serum and urine, possibly attributed to reduced bacterial growth rates. Additionally, as the effectiveness of PAC therapy strongly depends on phage-antibiotic concentration ratios, establishing appropriate dosages *in vivo* can be challenging (Ghali et al. 2023). Furthermore, even closely related phages may exhibit markedly different interactions with antibiotics, complicating optimal combination prediction (Carmen et al. 2020). Overcoming these challenges requires the establishment of PAC evaluation systems that simulate *in vivo* environments and a more detailed understanding of the molecular mechanisms underlying phage–antibiotic interactions.

##### Relationship between receptor selection and drug susceptibility

In phage cocktail design, target receptor selection is crucial, not only to ensure phage infection efficiency and resistance suppression, but also from an antibiotic interaction perspective. Recent reports have provided detailed molecular analyses of the relationship between phage target receptors and drug susceptibility.

Studies examining the combined effects of two phages recognizing different receptors (PP01 recognizing OmpC and SP15 recognizing FhuA) in *E. coli* O157 infection treated with fosfomycin found that combining SP15 and fosfomycin suppressed the emergence of resistant bacteria more effectively than the combination of PP01 and fosfomycin (Haeruman et al. 2024). This difference was attributed to the involvement of OmpC in fosfomycin permeability, as evidenced by an analysis using gene-deficient strains.

Additionally, in *P. aeruginosa*, extensive genomic deletions (in the BigD region) accompanying phage resistance acquisition can reportedly alter fluoroquinolone antibiotic susceptibility (Nakamura et al. 2021). The BigD region contains genes encoding drug efflux pumps, indicating potentially unexpected alterations in bacterial drug susceptibility through the acquisition of phage resistance. These findings indicate a trade-off between phage resistance and antibiotic susceptibility and provide new perspectives for phage cocktail design.

Furthermore, analysis of three phages against *P. aeruginosa* (phipa2, phipa4, and phipa10) provided novel insights into the relationship between phage resistance and drug susceptibility (Na et al. 2022). In particular, strains resistant to phages targeting type IV pili (T4P) (phipa2 and phipa4) exhibited reduced motility and biofilm formation capabilities and enhanced susceptibility to certain antibiotics. Conversely, strains resistant to phages targeting the O-antigen (phipa10) exhibited changes in cell membrane permeability and increased susceptibility to hydrophilic antibiotics. Notably, while phage-resistant *P. aeruginosa* mutants showed decreased biofilm formation capabilities, the opposite trend (increased biofilm formation) was observed in phage-resistant *K. pneumoniae* mutants (Yoo et al. 2024), thereby highlighting that the trade-off effects can vary depending on the bacterial species and the resistance mechanisms involved.

Another study demonstrated that structural changes in LPS that accompany resistance development to phage ΦX174 targeting the outermost part of the LPS R core in *E. coli* C strains can enhance susceptibility to antibiotics like chloramphenicol and gentamicin (Parab et al. 2025). The authors reported that bacteria in a ‘deep rough’ state with substantially shortened LPS owing to ΦX174 resistance exhibited high susceptibility to chloramphenicol. This phenomenon could be explained by the increased hydrophobicity and permeability of deep-rough-type LPS membranes compared with those of normal membranes, thereby facilitating the entry of small hydrophobic molecules, such as chloramphenicol.

These findings demonstrate the importance of considering the impact of target receptor selection on drug susceptibility when designing an effective phage cocktail. In particular, in phage cocktail design based on antibiotic combinations, a detailed examination of the effects of target receptors of each phage on drug uptake and efflux is necessary. Furthermore, combining phages targeting different receptors may reduce the risk of unexpected resistance. Future elucidation of the relationship between phage receptors and drug susceptibility across additional bacterial species will enable more effective phage cocktail designs.

### Cocktail design strategies

#### Sequential infection and simultaneous infection types

**Figure 6.**
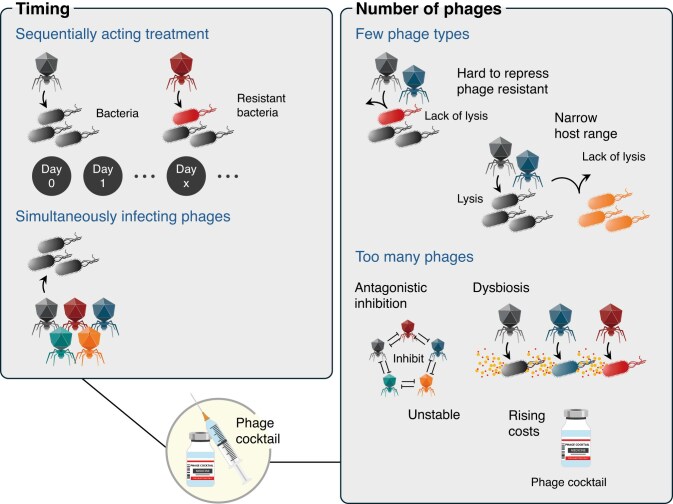
Strategic approaches for phage cocktail administration and composition. Two-panel illustration highlighting key strategic considerations in phage cocktail design. The left panel compares two primary administration approaches: sequential phage delivery and simultaneous administration of multiple phages. This timing choice represents an important design decision that can influence treatment efficacy. The right panel illustrates the importance of cocktail size optimization, showing that the number of phages included in a cocktail must be carefully determined to balance broad-spectrum coverage with potential stability issues and manufacturing complexity. Collectively, administration timing and cocktail component number represent critical parameters that must be optimized for each specific therapeutic application.

Beyond the criteria for constituent phages, cocktail design involves two major approaches from the perspective of chronologically administered phages: sequential and simultaneous administration (Fig. 6) (Smug et al. 2022). In sequential administration, one phage is designed to infect a resistant strain generated by another phage infection. By contrast, simultaneous administration involves multiple phages. A mathematical model analysis using PhREEPred (a web tool that simulates bacterial growth depending on phage cocktail composition, incorporating factors such as bacterial growth rate and initial density, multiplicity of infection, phage latent period, infectiveness, cocktail composition, and initial depolymerase concentration) suggested that sequential infection types suppress the emergence of resistant bacteria more effectively (Smug et al. 2022). This can be attributed to two primary mechanisms:

- Fitness costs associated with resistance acquisition: acquisition of resistance against one phage enhances susceptibility to another phage.
- Stepwise selection pressure: selection pressures acting at different times suppress resistant strain emergence completely.

However, recent research using phages with different host recognition mechanisms demonstrated the superiority of simultaneous over sequential administration in suppressing the emergence of resistant bacteria (Kim et al. 2024). These conflicting results suggest that the effectiveness of administration strategies may vary depending on the experimental conditions, phage and host characteristics, and resistance mechanisms. Accordingly, optimal administration strategies must be evaluated based on individual situations, necessitating further research in this field.

#### Cocktail size optimization

The number of phages included in cocktails must be determined by considering their effectiveness and balancing side effects. While multiphage cocktails are expected to increase the spectrum breadth and phage resistance suppression capacity, challenges other than increased manufacturing costs include potential antagonistic inhibition preventing efficient phage replication when multiple phages simultaneously infect bacteria, greater impact on microbiota, potential genetic transfer risks, and reduced stability. As a practical example, in the PhagoBurn trial, in which 12 types of phages were administered, cocktail instability exerted a negative impact on therapeutic efficacy. By contrast, stable effects were obtained upon treating *K. pneumoniae* and *E. coli* infections with 2‒10 and 8 phages, respectively (Jault et al. 2019, Nicolas et al. 2023, Kondo et al. 2023, Chen et al. 2024).

Beyond these considerations, the technical challenges of phage production represent critical practical constraints in cocktail size optimization. Achieving consistently high and reproducible titers for each constituent phage can be a complex and time-intensive process that significantly impacts manufacturing feasibility. For example, individual phages may exhibit substantial variability in production efficiency due to differences in burst size, latent period, and host bacterial growth requirements (Kaneko et al. 2023, Dai et al. 2023). Some phages may naturally produce relatively low titers even under optimized conditions, requiring disproportionate production efforts (Kok et al. 2023). Additionally, each phage may require distinct optimal culture conditions, making simultaneous large-scale production challenging (Chhibber et al. 2014, Lisac and Podgornik 2025).

From a manufacturing perspective, smaller cocktails may offer advantages including reduced production complexity, simplified quality control, and shorter manufacturing timelines. However, these considerations must be balanced against therapeutic requirements for adequate host range coverage and resistance suppression. Strategies to address these challenges include establishing production-optimized phage banks (“Utilization of Phage Banks”), developing standardized scalable platforms, and designing modular formulations where components are produced separately before final mixing.

### Manufacturing processes for clinical applications and intellectual property strategies

#### Standardization of manufacturing processes

The clinical application of phage cocktails requires the establishment of standardized manufacturing processes compliant with good manufacturing practices (GMP) (Brives and Pourraz 2020, Naureen et al. 2020). To establish a solid foundation for manufacturing processes, it is essential to create a workflow: first, construct a Master Seed Bank (MSB) that stores initially characterized strains under strict quality control, followed by the establishment of a Working Seed Bank, where strains for actual manufacturing are subcultured from the MSB and preserved. This two-stage system ensures genetic stability and quality consistency of phages used in manufacturing. Regular quality verification and update protocols must be established based on detailed evaluations of the characteristics of each phage.

Optimization of the cultivation processes is another crucial element. Host bacterial culture conditions must be standardized, and phage growth conditions (including MOIs, culture time, and temperature) must be optimized. Maintaining consistent quality during scale-up from laboratory to industrial manufacturing remains an important manufacturing challenge. This transition requires addressing multiple technical considerations: ensuring uniform phage titers across production batches, maintaining purification efficiency at larger volumes, and balancing production costs with GMP compliance requirements (Mohammadi et al. 2025). Bioreactor-based cultivation systems enable controlled environmental conditions and reproducible phage yields, although optimization of parameters such as MOIs, harvest timing, and aeration rates must be tailored to specific phage-host systems. During purification processes, endotoxin removal by ultrafiltration or chromatography, sterility assurance by filtering through a 0.22-µm filter, and controlling impurities such as host-derived proteins and DNA, are necessary (Naureen et al. 2020). For large-scale manufacturing, scalable purification methods such as anion-exchange chromatography combined with enzymatic treatments, tangential flow filtration, and continuous processing technologies are essential to achieve adequate removal of endotoxins, proteins, and host cell DNA while maintaining high phage recovery rates, particularly for preparations intended for intravenous administration where regulatory agencies strictly control impurity levels (Mohammadi et al. 2025, Saavedra et al. 2025).

#### Quality control and stability evaluation

Quality control of cocktail formulations requires evaluation at both the individual phage and overall cocktail levels. The PhagoBurn trial demonstrated the importance of quality control. In this trial, while individual phages remained stable at concentrations exceeding 1 × 10^9^ PFU/ml for over 24 months, the overall titre decreased from 1 × 10^9^ PFU/ml to 1 × 10^4^–10^5^ PFU/ml after mixture as a cocktail (Jault et al. 2019).

Based on the above experiences, long-term storage tests at -80°C, 4°C, and room temperature, along with stress tests (effects of pH, temperature, and light), are considered standard requirements in formulation stability evaluations. To prevent titre reduction after cocktail preparation, evaluation of individual phage stability, detailed analysis of interactions, formulation stabilization (e.g. adding protective agents), and establishment of appropriate storage conditions are essential.

#### Frameworks for standardization

To address the manufacturing and quality control challenges exemplified by the PhagoBurn trial, several practical frameworks can guide the standardization of phage cocktail production.

- *Tiered Quality Control System*

A risk-based, tiered approach may be most practical for phage cocktail standardization (Malik et al. 2023). Critical Quality Attributes (CQAs) should be identified and monitored at three levels: (i) individual phage level (titer, purity, genomic stability), (ii) cocktail formulation level (phage ratio maintenance, overall titer, pH, sterility), and (iii) final product level (stability over time, absence of endotoxins, maintenance of biological activity). The PhagoBurn experience suggests that stability testing should occur not only for individual phages but also for the final mixed cocktail under intended storage conditions, with defined acceptance criteria established before manufacturing scale-up (Jault et al. 2019). Mapping Critical Process Parameters to CQAs is essential to ensure quality and consistency throughout phage drug substance and drug product development (Malik et al. 2023).

- *Process Analytical Technology Implementation*

Real-time monitoring systems adapted from biopharmaceutical manufacturing could enhance phage production consistency and reduce batch-to-batch variability (Malik et al. 2023, Mohammadi et al. 2025). Process Analytical Technologies (PAT) enable better process monitoring and control, improving both process robustness and productivity in achieving high phage titers (Malik et al. 2023). Potential PAT applications include: inline optical density monitoring to determine optimal harvest timing, flow cytometry for rapid phage enumeration during production, and quantitative PCR for monitoring phage genome copy numbers (Brussaard 2009, Refardt 2012). These technologies may enable more precise process control and earlier detection of batch-to-batch variability compared with traditional plaque assays alone.

- *Modular Manufacturing Approach*

A modular strategy, where individual phages are produced, purified, and quality-tested separately before final cocktail assembly, may offer several advantages. This approach allows: (i) independent optimization of production conditions for each phage, (ii) flexible cocktail composition adjustments based on emerging resistance patterns or individual phage batch failures, (iii) reduced risk of complete batch loss, and (iv) simplified troubleshooting when quality issues arise. Single-use technologies are increasingly becoming the preferred manufacturing option, offering benefits for both phage bioprocess development at the engineering run research stage and for final manufacture of phage drug substance (Würstle et al. 2024). Individual phage components could be stored as frozen stocks at defined concentrations, with final cocktail assembly occurring closer to the point of use to minimize stability concerns.

- *Standardized Stability Testing Protocols*

Based on lessons from the PhagoBurn trial, comprehensive stability testing protocols should include: (i) accelerated stability studies under stress conditions (elevated temperature, pH extremes, light exposure) to predict shelf life, (ii) real-time stability monitoring of cocktails at intended storage conditions over extended periods (e.g. individual phages for ≥24 months and mixed cocktails for ≥6 months, although optimal timeframes require further validation), (iii) evaluation of multiple parameters beyond titer (maintenance of phage ratios, biological activity against target strains, endotoxin levels), and (iv) stability testing in relevant biological matrices (e.g. wound exudate, urine, serum) when applicable (O’Connell et al. 2022, Malik et al. 2023). Characterization of physiochemical properties (size, charge, etc.), buffer pH and osmolality, compatibility with regulatory-approved excipients, storage stability considerations (packaging, temperature, humidity), and ease of manufacturability should be incorporated into formulation development (Malik 2021). Establishment of defined failure criteria and contingency plans (e.g. reformulation strategies) should be incorporated into manufacturing protocols before clinical implementation.

- *Collaborative Standardization Initiatives*

Given the nascent state of phage therapy manufacturing, collaborative efforts may accelerate standardization (Anastassopoulou et al. 2025). Initiatives such as the development of reference materials, inter-laboratory proficiency testing programmes, and shared databases of production parameters for well-characterized phages could provide benchmarks for quality (Maffei et al. 2021, Humolli et al. 2025, Cook and Hynes 2025). Collaboration between academia, industry, and regulatory agencies is essential to establish consistent standards, foster innovation, and bridge the gap between academic research and commercial applications.

These frameworks are not mutually exclusive and may be most effective when implemented in combination, adapted to the specific characteristics of individual cocktails and intended clinical applications.

#### Patenting strategies

The intellectual property protection of phage cocktails is a crucial element of commercialization. Recently, trends from the United States regarding Mayo and Myriad decisions have restricted the patenting of natural laws and natural products, affecting the patent eligibility for phage therapy. This raises concerns that natural phage lytic activity utilization may be denied patent eligibility as ‘application of natural law’ and that isolated phages may be considered ‘natural products’ (Yang et al. 2024). However, patent systems differ considerably by region, necessitating strategies to be adapted to the legal system of each region. In Europe, microbiological processes and their products are patentable, while in Australia, biological materials isolated from natural environments may qualify as patent subjects (Yang et al. 2024, 144). Japan allows patents, even for individual phages.

Specific patent acquisition strategies include patenting artificially modified phages or cocktails combining multiple phages (Yang et al. 2024). Since 2018, patents with a sequence similarity threshold of ≥97% have been approved as standard. However, standardizing the calculation methods, including alignment algorithm selection and gap penalty settings, remains challenging. Comprehensive protection strategies that combine therapeutic applications and specific usage methods have also been adopted (Yang et al. 2024).

Complementary patent protection measures are also important. These include experimental use exception provisions permitting research-purpose use (Oliveira et al. 2015), trade secret protection of know-how, and obtaining market exclusivity through regulations (Todd 2019). Recent trends have shown an increasing number of patents related to biochemical modifications and binding, such as phage encapsulation in polymer microcapsules or binding to particles to enhance performance (Yang et al. 2024).

Establishing collaborations between public healthcare systems and industry-academia-government cooperation frameworks is also a pivotal element in the practical implementation of phage cocktails. To address these challenges, future efforts should focus on constructing more flexible, adaptive legal frameworks and harmonizing international patent environments.

## Clinical applications and insights gained

This section introduces the implementation of the phage cocktail design principles discussed in “Design Principles of Phage Cocktails” in clinical and application settings, with case examples. This analysis clarifies the differences between theory and practice and identifies essential points for practical guidelines in phage cocktail design.

### Applications for priority drug-resistant bacterial pathogens

#### A. baumannii infections: application of personalized cocktails

A 68-year-old male with necrotizing pancreatitis caused by MDR *A. baumannii* infection was administered surface (individualized) treatment using personalized phage cocktails (Schooley et al. 2017). Based on the clinical strains (TP1) isolated from the patient, phage screening was conducted at multiple research institutions to select and combine multiple phages obtained from environmental samples. The selection criteria emphasized the breadth of the host range and lytic activity. Accordingly, four phages from the Navy library (AB-Navy1, AB-Navy4, AB-Navy71, AB-Navy97) demonstrating specific lytic activity against the TP1 strain were adopted into the initial ‘ΦIV’ cocktail.

During treatment, local administration via an intraperitoneal catheter (~10^9^ PFU/dose of ΦPC cocktail every 6–12 hours for 18 weeks) was combined with intravenous administration. The initial intravenous cocktail (ΦIV, 5 × 10^9^ PFU/dose) was administered for 16 weeks, with the core period of combination therapy lasting ~59 days. These initial cocktails were designed by considering the complementary actions of multiple phages to suppress the evolution of resistant strains. When resistant strains (TP3) emerged during treatment, a new phage (AbTP3φ01) was incorporated with one of the original phages (AB-Navy71) to construct ‘ΦIVB’. This updated cocktail (ΦIVB) was intravenously administered at 5 × 10^9^ PFU for 2 weeks. The iterative adaptation of the cocktail in response to emerging resistance represents an important practical example of a design that utilizes phage-resistant strains (“Design Utilizing Phage-Resistant Strains”). This case demonstrates the potential of personalized phage cocktails as an important treatment option for MDR bacterial infections.

A follow-up report on this case provided a genome analysis of the nine phages used and three *A. baumannii* strains isolated before and during phage therapy (Liu et al. 2022). Initial treatment utilized two cocktails: the ΦPC cocktail administered intraperitoneally and the ΦIV cocktail administered intravenously. Both contained four T4-like myophages; however, genome analysis revealed that some phages within the cocktails were genetically identical and were classified into only two groups based on tail fibre protein sequences. This genetic similarity likely caused the emergence of phage-resistant bacteria merely 2 days after treatment initiation.

During treatment, AbTP3φ01, a Fri1-like podophage from a different lineage, was isolated to address newly emerged phage-resistant strains (TP3) and combined with one phage from the ΦIV cocktail to create a new cocktail (ΦIVB). This cocktail demonstrated efficacy against the resistant bacteria. Genome analysis revealed that while the myophage group could recognize and infect bacterial capsules, AbTP3φ01, a podophage morphologically distinct from the myophages in the initial cocktails, mediated infection through distinct mechanisms. Genomic analysis revealed that its tail spike protein possesses depolymerase activity, specifically targeting the K116 capsular polysaccharide of the *A. baumannii* TP1 strain. Furthermore, genome comparison of strains isolated before (TP1) and during treatment (TP2 and TP3) revealed that they were mutant variants of identical clones, with phage resistance primarily attributable to mutations in the glycosyltransferase gene (gtr76) involved in capsule synthesis. Additionally, the same mutations were observed in phage-resistant mutant strains created in the laboratory using the TP1 strains, demonstrating that studies exploring resistance mechanisms *in vitro* can predict results relevant to actual clinical scenarios.

The discussed case demonstrates the importance of combining phages recognizing different receptors to suppress the emergence of resistant bacteria and the effectiveness of including ‘guard’ phages in cocktails to address phage-resistant bacteria. The importance of preliminary genome analysis in phage cocktail design was also emphasized.

#### *P. aeruginosa* infections: lessons from clinical evaluation trials

The PhagoBurn trial, a randomized controlled trial, represents an important turning point in phage cocktail clinical evaluation (Jault et al. 2019). This trial formulated a cocktail (PP1131) combining 12 phages, primarily intended to acquire a broad host range (prêt-à-porter approach). As the first phage cocktail to be manufactured according to GMP standards in Europe, it provides important insights into manufacturing and stability.

Nevertheless, substantial challenges were encountered during the cocktail manufacturing process. While individual phages remained stable at concentrations exceeding 1 × 10^9^ PFU/ml for over 24 months, the overall titre decreased from 1 × 10^9^ PFU/ml to 1 × 10^4^-10⁵ PFU/ml after mixture as a cocktail. According to the results of the stability tests, the cocktail titre decreased by ~1000-fold within 15 days of cocktail preparation. This experience demonstrates the importance of standardizing the manufacturing process (“Standardization of Manufacturing Processes”) and stability evaluation (“Quality Control and Stability Evaluation”). Ultimately, the authors were forced to administer the cocktail preparation at concentrations (10² PFU/ml) considerably lower than expected (10^6^ PFU/ml), which resulted in delayed onset of effects compared with standard treatment groups and premature termination of the trial.

Conversely, individualized phage therapy (a sur-mesure approach) was successful against MDR *P. aeruginosa* infection with vertebral abscesses (Ferry et al. 2022). Through academic collaboration spanning Switzerland, Belgium, and France, patient-specific cocktails comprising three phages (vB_PaeP_4029, vB_PaeP_4032, and vB_PaeP_4034) were developed and administered against vertebral abscesses caused by strains resistant to existing commercial phages. The treatment involved a combination of comprehensive approaches, including surgical procedures, antibiotic therapy, and local and intravenous cocktail administration. Despite the emergence of phage-resistant strains during treatment, infection was ultimately controlled. This case demonstrates the importance of rapidly developing and administering patient-specific phage cocktails when existing phages are ineffective, as well as the value of academic cooperative frameworks for this purpose. In addition, this case provides valuable insights into phage therapy strategies in clinical settings, including combining different administration routes (local and intravenous) and combination with antibiotics and surgical procedures.

#### *S. aureus* infections: importance of pre-evaluation and monitoring

A comprehensive retrospective observational study of 114 phage therapy cases among 100 patients has reported on phage cocktail clinical applications (Pirnay et al. 2024). This study analysed phage therapy results for refractory infections across 35 hospitals in 12 countries based on a special individualized approach by a Belgian consortium, known as ‘Magistral Phage’, with *S. aureus* infections constituting 39% (39/100) and *P. aeruginosa* infections identified as the most prevalent at 49%. Cocktails primarily based on *Staphylococcus* phage ISP were used against *S. aureus* and were selected based on the diversity of lytic activity and target receptor differences. This approach represents a practical example of the principle of ‘combining phages targeting different receptors’ discussed in “Strategies for Combining Different Phages”.

The clinical outcomes revealed clinical improvement in 77.2% of patients and target bacterial eradication in 61.3% of patients. Statistical analysis demonstrated that phage therapy combined with antibiotics resulted in significantly higher target bacteria eradication rates than phage monotherapy (odds ratio 0.3; 95% confidence interval 0.127‒0.749). In particular, *S. aureus* phage ISP showed synergistic effects with clindamycin, vancomycin, and ceftaroline. These findings provide important clinical evidence supporting ‘strategies combining phages and antibiotics’ discussed in “Strategies for Combining Phages and Antibiotics”.

Notably, ‘pre-adaptation’ of phages has been implemented in certain cases to enhance their therapeutic effects. This process involves adapting phages to patient-specific bacterial strains through continuous passage, representing a practical example related to ‘design utilizing phage-resistant strains’ from “Design Utilizing Phage-Resistant Strains”.

The emergence of phage-resistant strains was confirmed in 7 of 16 cases (43.8%). However, some phage-resistant strains demonstrate re-sensitization to antibiotics and reduced toxicity, potentially contributing to treatment success. Additionally, while phage-neutralizing antibodies emerged 6‒35 days later in 5 of 13 cases (38.5%) receiving invasive administration (intravenous and/or intralesional), most achieved clinical improvement and targeted bacterial eradication.

Several notable examples comparable to the above phenomenon have been documented in comprehensive reviews (Plumet et al. 2022), including a study demonstrating that a three-phage cocktail exhibited superior efficacy compared with linezolid monotherapy in diabetic foot infection models (Albac et al. 2020).

These studies demonstrate that three principles are clinically effective in phage cocktail design: (i) combining phages targeting different receptors, (ii) combining phages with antibiotics, and (iii) phage adaptation when necessary. Moreover, successful phage therapy requires detailed pretreatment evaluation (phage sensitivity testing and phage-antibiotic interaction evaluation) and continuous monitoring during treatment (resistant strain emergence and neutralizing antibody development).

#### *E. faecium* infections: impact of immune response on treatment efficacy

In phage therapy against recurrent *E. faecium* bacteraemia unresponsive to antibiotic combination therapy, treatment combining a single phage (Φ9184) with multiple antibiotics resolved bacteraemia persisting for 26 days within 24 hours, with temporary clinical improvement, although bacteraemia recurrence was observed during treatment (Stellfox et al. 2024). In response, transitioning to two phage cocktails (Φ9184 and ΦHi3) suppressed bacteraemia for 4 months, improving the patient’s quality of life. Interestingly, ~5 months after treatment initiation, neutralizing antibodies against phages emerged, substantially reducing the activity of the phage cocktails. Serological analysis revealed that this neutralization was mediated by a significant increase in phage-specific IgG antibodies, which correlated with the timing of treatment failure. This should be noted as a potential contributor to treatment failure. Additionally, metagenomic analysis demonstrated that phage therapy reduced the intestinal *E. faecium* and vancomycin-resistant enterococci (VRE) populations. This study suggests that phage cocktails containing multiple phages are more effective than single phages, while emphasizing the importance of phage administration plans that consider host immune responses for sustained therapeutic effects.

A study using mice also provided important insights into the selection of phages for therapeutic application by comprehensively evaluating the efficacy of phage cocktail therapy against VRE intestinal colonization and phage-specific immune response influences (Berkson et al. 2024). The cocktail, designed by combining phages that recognize different receptors (“Receptor-Based Approaches”), comprised five phages belonging to *Myoviridae* and *Siphoviridae* families. Administration of the designed cocktail reduced VRE numbers in mouse faeces by ~2 log CFU/g. However, two cocktail administrations (at 4-week intervals) induced neutralizing antibodies against all phages, with stronger neutralizing antibody responses observed against *Myoviridae* family phages. Additionally, antibody-binding patterns differed by phage family, with entire particles targeted in *Myoviridae* family phages compared with the targeting of primarily the tail parts in *Siphoviridae* family phages. Furthermore, the induction of this immune response promotes phage clearance from tissues, significantly attenuating the effectiveness of the second phage therapy against VRE intestinal colonization. This study demonstrates the importance of considering the influence of phage-specific immune responses to ensure the long-term effectiveness of phage therapy. Particularly, the necessity of using phages with different immunogenicity profiles has been suggested in phage therapies against recurrent infections. In addition to humoral immune activation through neutralizing antibody production, phage elimination by the mononuclear phagocyte system represents another critical factor that can significantly affect therapeutic efficacy. Macrophages and other phagocytic cells can rapidly clear phages from circulation, especially after opsonization by complement proteins or antibodies. This clearance mechanism can reduce the effective concentration of phages at infection sites and limit therapeutic duration, potentially necessitating repeated administrations or the use of phage preparations with modified surface properties to extend circulation time (Kurzepa et al. 2009).

#### *E. coli* infections: optimization of administration routes

A phase II clinical trial examined a six-phage cocktail (LBP-EC01) against *E. coli* urinary tract infections (Kim et al. 2024). The cocktail comprised three phages enhanced with CRISPR-Cas3 systems, in addition to combining phages that recognize different receptors (“Receptor-Based Approaches”). This cocktail demonstrated activity against 96% of 356 uropathogenic *E. coli* strains.

This trial compared the intravenous and intraurethral administration of the phage cocktail. The combination of intraurethral (2 × 10^12^ PFU) and low-dose intravenous administration (1 × 10^10^ PFU) demonstrated the best tolerability and maintenance of phage concentration. A rapid reduction in urinary *E. coli* was observed 4 hours after treatment initiation, with a microbiological cure confirmed in 14 of the 16 evaluable cases (88%) by day 10. Treatment effects were also observed in 9 of 11 patients (82%) infected with trimethoprim-sulfamethoxazole-resistant strains.

This trial suggests that, beyond phage cocktail design, optimizing administration routes substantially influences therapeutic effects. In particular, maintaining sufficient phage concentrations at the infection sites is pivotal for clinical efficacy. These findings demonstrate the importance of the formulation and administration methods discussed in “Standardization of Manufacturing Processes” and “Quality Control and Stability Evaluation".

Another report described a four-phage cocktail administered for the successful treatment of recurrent urinary tract infections caused by extended-spectrum β-lactamase-producing *E. coli*, rationally designed based on the principles discussed in “Strategies for Combining Different Phages”, aiming for particularly effective combinations (Terwilliger et al. 2021). This cocktail included three types of phages with distinct properties. First, it contained phages with mucolytic properties that promote bacterial death under physiological conditions. Second, the cocktail included phages that bind to heparan sulfate proteoglycans present on human mucosal surfaces. Third, phages were obtained through directed evolution targeting the major pathways of phage resistance to circumvent phage defence mechanisms of *E. coli*.

This cocktail was administered intravenously for 2 weeks, along with the concurrent administration of ertapenem for 6 weeks. The patient tolerated the treatment without experiencing adverse events, and the symptoms disappeared. Interestingly, follow-up examination 6 weeks after treatment completion revealed asymptomatic bacteriuria, but the isolated strains, which were closely related to the original strains, carried mutations in proteins involved in adhesion and invasion and maintained susceptibility to phage cocktails, similar to the causative bacteria.

These findings underscore: (i) the effectiveness of cocktail designs combining phages with different functions, (ii) synergistic effects with antibiotics, and (iii) the possibility that altering bacterial characteristics (transition to less pathogenic strains) rather than complete pathogen elimination may also yield desirable clinical outcomes. This represents an important clinical example supporting the perspective that resistance mechanisms *in vivo* may differ from those predicted *in vitro* owing to microenvironmental influences at infection sites and immune system effects.

These findings demonstrate the practical importance of the theoretical design criteria discussed in “Design Principles of Phage Cocktails” and indicate that an effective phage cocktail design requires detailed pre-evaluation and continuous monitoring (Table 2).

**Table 2.** Representative clinical trials of individualized phage therapy.

Study	Pathogen	Infection site	Phage(s)	Administration route (cocktail)	Dosage	Duration	Outcome	Notable findings
Schooley et al. 2017	*A. baumannii* (MDR)	Disseminated (pancreatic abscess, etc.)	9 phages total (e.g. AB-Navy1,4,71,97; AbTP3Φ01)	Local (ΦPC) + IV (ΦIV, later ΦIVB)	Local: ∼10^9^ PFU/instillation IV: 5 × 10^9^ PFU/dose	Total: ∼18 weeks Core Combo: ∼59 days	Recovery	Iterative cocktail design to counter resistance. Podophage effective against capsule-deficient mutants.
Jault et al. 2019 (PhagoBurn)	*P. aeruginosa*	Burn wound infection	PP1131 cocktail (12 phages: Podoviridae/Myoviridae)	Topical (via alginate dressing)	∼1 × 10^2^ PFU/ml (actual delivered dose, due to stability issues)	7 days	Slower bacterial reduction compared with standard of care	Critical reduction in phage titer after manufacturing led to subtherapeutic dosing. Highlights the importance of phage cocktail stability and *in vitro* susceptibility testing (phagogram).
Ferry et al. 2022	*P. aeruginosa* (pandrug-resistant)	Spinal abscess (spondylodiscitis)	vB_PaeP_4029, 4032, 4034	Local (intra-lesional) + IV (sequential)	Local: 10^6^ PFU/mL (7 ml) IV: 10^6^ PFU/ml (30 ml daily)	Local: 2 doses (surgeries) IV: 21 days	Clinical cure (infection resolved)	Sequential local + IV administration. Small Colony Variant (SCV) emerged but remained phage-sensitive.
Stellfox et al. 2024	*E. faecium* (VRE)	Bacteraemia	Φ9184 (siphovirus), later ΦHi3 (siphovirus) added (sequential)	IV + oral	Initial (Φ9184): 1 × 10^9^ PFU/dose, TID cocktail (Φ9184+ΦHi3): 2 × 10^9^ PFU/dose, TID → BID	∼6 months of active therapy	Initial clearance and 4-month remission, then recurrence	Neutralizing anti-phage IgG antibodies emerged after ∼5 months, abrogating phage activity. No phage resistance developed in clinical isolates.
Kim et al. (ongoing)	*E. coli*	Uncomplicated UTI	LBP-EC01 (CRISPR-Cas3-enhanced 6-phage cocktail)	Intraurethral + IV (concurrent)	Intraurethral: 2 × 10^12^ PFU (days 1–2) IV (Group A): 1 × 10^10^ PFU daily (days 1–3) + Oral TMP-SMX	IV: 3 days Local: 2 days	Rapid reduction of *E. coli* and symptom resolution by day 10 in evaluable patients	First-in-human trial of a CRISPR-enhanced phage. IV dose-dependent systemic exposure and tolerability. Demonstrates the engineered phage approach.
Terwilliger et al. 2021	*E. coli* (ESBL-producing)	Recurrent UTI/prostatitis	HP3, HP3.1, ES17, ES19 (rationally designed 4-phage cocktail)	IV	1 × 10^9^ PFU/dose, twice daily + IV ertapenem	2 weeks (phage), 6 weeks (antibiotic)	Symptom resolution; asymptomatic bacteriuria with attenuated strain at follow-up	Neutralizing antibodies emerged by week 2. Rationally designed cocktail included a mucolytic phage and an evolved ‘guard’ phage. Microbial succession without full eradication led to clinical cure.

Abbreviations: IV, intravenous; UTI, urinary tract infection.

### Important insights and prospects from clinical applications

Multiple crucial insights into the effectiveness of phage cocktail therapies have been derived through clinical application. First, as demonstrated by cases of *A. baumannii* and *S. aureus* infection, detailed pre-evaluation and continuous monitoring during treatment are important success elements. In cases that implemented pre-evaluation, 77.2% clinical improvement was achieved, and monitoring during treatment enabled appropriate responses to the emergence of resistant strains (Pirnay et al. 2024).

Second, the PhagoBurn trial and *E. coli* infection clinical trials revealed that formulation stability and selection of the administration route could substantially influence therapeutic effects. Appropriate formulation and administration route selection are essential for maintaining sufficient phage concentrations at the infection sites. Additionally, as shown in *E. faecium* studies, host immune responses against phages are key factors influencing their therapeutic effects. In particular, differences in immunogenicity by phage family should be considered in cocktail design.

These insights demonstrate that effective phage cocktail development requires three elements: (i) detailed pre-evaluation and continuous monitoring; (ii) appropriate formulation and administration route optimization; and (iii) consideration of the host immune response. Bridging the gap between personalized approaches and GMP standard-based manufacturing standardization is likely an important aspect of future practical implementation.

## Future prospects and challenges

This section organizes the unresolved issues in phage cocktail research and applications, along with future research directions. In particular, the discussion focuses on the establishment of evaluation criteria, overcoming technical challenges, and future possibilities.

### Systematization of evaluation criteria

#### *Establishment of in vitro* evaluation systems

Currently, the evaluation of initial lytic activity using metrics such as the VI and host range assessment is common in phage cocktail evaluation (Storms et al. 2020). However, these methods are biased toward short-term evaluation of lytic effects and encounter the following challenges:

- *Lack of long-term effect evaluation criteria*

The lack of long-term evaluation criteria is problematic. Quantifying sustained antibacterial effects in continuous culture systems can enable evaluation under conditions closer to actual use environments. Additionally, evaluation of sustained antibacterial effects under static conditions is important for predicting long-term effects, including the emergence of resistant bacteria. For instance, turbidity measurements over 72 hours have been used to evaluate the emergence of resistant bacteria and the effectiveness of phage cocktails (Kim et al. 2024). Furthermore, the evaluation of anti-biofilm activity represents an important aspect of long-term effectiveness assessment, which is discussed in detail in “Evaluation of Anti-biofilm Activity of Phage Cocktails”.

- *Evaluation of cocktail-specific interactions*

When combining multiple phages, appropriate evaluation of phage–phage interactions is necessary. In particular, quantitative methods for evaluating synergistic (Tanji et al. 2004, Bai et al. 2019, Yang et al. 2020, Chen et al. 2024) and antagonistic (Hofer et al. 1995, Bourdin et al. 2014, Bondy-Denomy et al. 2016, Silpe et al. 2019) effects are required. Individual evaluation of the contribution of each phage within cocktails is essential to select optimal combinations. For example, some cocktails demonstrate narrower host ranges than the sum of the individual phage host ranges, possibly because of competition between phages sharing identical receptors or competition for limited cellular resources (Bourdin et al. 2014). Additionally, inhibition of superinfection by lysogenic phages may contribute to host resistance in some cases (Hofer et al. 1995, Bondy-Denomy et al. 2016). These findings highlight the importance of individually evaluating the contribution of each phage to the cocktail.

#### Establishment of *in vivo*evaluation systems

For *in vivo* evaluations, such as in animal models and clinical settings, a comprehensive assessment from both efficacy and safety perspectives is necessary. Current evaluation systems face the following challenges:

- *Standardization of efficacy evaluation*

Unlike conventional antibiotics, phages are self-replicating biological agents, making their pharmacokinetics extremely complex. In particular, phage accumulation is reportedly promoted owing to the presence of bacteria at the infection site compared with healthy tissues (Nang et al. 2023). Additionally, because phage distribution in the body varies considerably with the administration route, it is crucial to select optimal administration routes according to the infection site.

- *Systematization of safety evaluation*

For phage safety evaluation, a detailed examination of interactions with the immune system is necessary. For example, neutralizing antibody production dynamics can substantially influence the therapeutic effects of certain phages (Berkson et al. 2024). Some phages may induce natural immune responses via TLR3, potentially inhibiting bacterial clearance by suppressing TNF production (Sweere et al. 2019). Unexpected immune responses, such as the promotion of neutrophil migration via tumour-associated macrophage activation, have also been reported (Eriksson et al. 2009). Therefore, a systematic evaluation of the following items is necessary.

- Evaluation of phage-specific neutralizing antibody production dynamics
- Evaluation of natural immune responses via pattern recognition receptors
- Quantitative measurement of inflammatory cytokines
- Safety confirmation through histological evaluation
- *Phage cocktail-specific challenges*

*In vivo* evaluation of phage cocktails must consider potential differences in the dynamics of each phage in the cocktail. For example, the concentration of one phage in the blood may rapidly decrease, whereas another phage may remain in circulation for a prolonged period. Establishing systems capable of identifying and quantifying individual phages within cocktails is essential for evaluating differences in individual phage pharmacokinetics. Potential phage–phage interference phenomena should also be considered.

- *Immune response diversity*

Because phage cocktails contain multiple phages, host immune responses may differ for each phage. Particularly, when repeated administration is implemented for recurrent diseases, neutralizing antibodies may be produced against specific phages, potentially altering the cocktail-mediated effect over time. Temporal measurement of antibody titres against individual phages is necessary to evaluate the immune response diversity.

- *Long-term monitoring of resistant bacteria emergence*

*In vivo*, owing to microenvironmental influences at infection sites and immune system effects, resistance mechanisms distinct from those predicted *in vitro* may be expressed. Therefore, long-term monitoring of resistant bacteria during and after treatment and a detailed analysis of the characteristics of emerging resistant bacteria are important. Cases in which resistant bacteria emerging *in vivo* simultaneously acquire resistance to multiple phages within the cocktail are particularly noteworthy.

- *Establishing appropriate standardized controls*

Establishing appropriate control groups is pivotal for the *in vivo* evaluation of phage cocktails. In addition to comparing single phages with cocktails, establishing groups excluding specific phages from cocktails should be considered to determine the extent to which each phage contributes to the therapeutic effects. Direct comparisons with standard antibiotic treatments and evaluation of combination effects are also important.

#### Evaluation of anti-biofilm activity of phage cocktails

The evaluation of phage cocktail efficacy against bacterial biofilms represents a critical gap in current assessment methodologies, particularly given that most human bacterial infections are associated with biofilm formation. Biofilms confer high resistance to antibiotics and host immune defences, making phage therapy—and especially phage cocktails—potentially valuable therapeutic tools due to their ability to penetrate biofilms, produce depolymerizing enzymes, and act synergistically with antibiotics (Dsouza et al. 2024, Khan et al. 2025).

*In vitro* biofilm evaluation methods for phage cocktails include several complementary approaches. Commonly used assays include viable cell counting (CFU determination), metabolic activity staining such as Alamar Blue and soluble tetrazolium dyes, and biomass quantification using crystal violet staining (Latka and Drulis-Kawa 2020). Metabolic activity staining enables more quantitative assessment than conventional viable cell counts alone. However, biomass determinations can be both inaccurate and imprecise, necessitating characterization using multiple methods such as combining biomass staining with CFU counts (Abedon et al. 2021).

A critical consideration in biofilm evaluation is distinguishing between phage enzymatic activity and phage bacteriolytic activity. Phages encoding extracellular polymeric substance depolymerases can disrupt biofilms without associated phage-induced bacterial lysis (Vukotic et al. 2020, Bseikri et al. 2025). The presence of depolymerizing activity can be detected macroscopically through the formation of translucent halos surrounding plaques on bacterial lawns, with halo enlargement resulting from enzyme diffusion independently of phage replication (Vukotic et al. 2020).

Phage cocktails demonstrate superior anti-biofilm activity compared with single phages through multiple mechanisms. Cocktail approaches can delay the emergence of phage-resistant bacteria and provide complementary antimicrobial elements, such as combinations of depolymerase-producing and non-depolymerase-producing phages (Chang et al. 2022). Studies have shown that phage cocktails can achieve substantial biofilm reduction (at least two-fold within 24 hours) against strong biofilm-producing isolates, with some achieving 2–3 log reductions in biofilm-associated viable bacterial count (Vukotic et al. 2020, Manohar et al. 2024).

Key experimental considerations for *in vitro* biofilm evaluation include: (i) distinguishing biofilm removal from prevention of new biofilm growth through zero-time-point determinations, (ii) employing kinetic rather than solely end-point analyses, (iii) testing multiple dosing regimens and treatment durations, and (iv) characterizing biofilm presence using multiple methods rather than relying on a single assay (Abedon et al. 2021). Without comprehensive anti-biofilm evaluation methodologies, the translational potential of phage cocktail therapy remains significantly limited.

### Technical challenges

Various technical challenges, ranging from manufacturing to quality control, must be overcome for the practical implementation of phage cocktails.

- *Standardization of manufacturing processes*

The establishment of GMP-compliant manufacturing processes is one of the most important challenges in clinical application. Establishing endotoxin management standards and developing quality control methods during mass production are essential for guaranteeing product safety and efficacy (Bretaudeau et al. 2020, Suleman et al. 2025). Insufficient quality control in manufacturing not only compromises patient welfare but may potentially set back phage therapy field development by years.

- *Establishment of quality control*

Maintaining the constituent phage ratios within cocktails is crucial for ensuring product consistency. In particular, optimizing storage conditions (temperature, pH, and buffer composition) and ensuring quality consistency are essential for clinical applications (Suleman et al. 2025, Faltus 2024). When quality control is insufficient, patients may receive phage preparations of different qualities, making it difficult to predict the therapeutic effect.

### Future outlook

Phage cocktail research anticipates significant developments in both the introduction of new technologies and the establishment of a foundation for practical implementation.

- *Utilizing new technologies*

A mathematical analysis of PBIN and artificial intelligence (AI) applications is expected to enable a more efficient phage cocktail design. Methods for quantifying the importance of individual phages (Díaz-Galián et al. 2022) and predicting optimal combinations through exhaustive searches (Menor-Flores et al. 2022) have been reported. Host specificity prediction using random forest models with PBIN data could be applied in effective cocktail designs against clinical isolates (Keith et al. 2024). Furthermore, high-accuracy prediction (AUROC 86%) of phage–host interactions across the entire *Escherichia* genus using only genomic information has been demonstrated (Gaborieau et al. 2024). This technology is expected to play a vital role, particularly in cocktail host range prediction.

Synthetic biology approaches have introduced new possibilities for cocktail design. For example, technologies that modify *E. coli* phage genomes using budding yeast have successfully generated phages targeting *Yersinia* and *Klebsiella* by replacing the host recognition components with those from phages specific to these bacteria (Ando et al. 2016). Additionally, the development of modified phages incorporating CRISPR-Cas mechanisms enables more specific targeting and suppression of the emergence of resistant bacteria (Gencay et al. 2024). These technologies are expected to play a pivotal role in optimizing phage cocktail components.

- *Foundation establishment toward practical implementation*

Establishing evaluation criteria based on regulatory science is an important step towards practical implementation (Abedon et al. 2021). In particular, the development of safety evaluation criteria for the use of modified phages as cocktail components is required. Additionally, the construction of rapid efficacy evaluation systems using AI (Menor-Flores et al. 2022) could serve as a powerful tool for clinical implementation. The establishment of implementation strategies for personalized medicine (Abedon et al. 2021) is also expected to lead to the development of more effective therapies.

## Conclusions

This review comprehensively examines phage cocktail design criteria, particularly from the perspective of suppressing the emergence of phage-resistant bacteria. To date, research has established that combining phages targeting different receptors and combining with antibiotics represents an effective therapeutic strategy. However, as demonstrated by the PhagoBurn trial and the large-scale analysis by Pirnay et al. 2024, challenges remain in ensuring cocktail stability and in suppressing the emergence of resistant bacteria. Future expectations include overcoming these challenges by utilizing innovative technologies such as AI and synthetic biology, and by establishing more systematic evaluation systems. In particular, the realization of highly individualized cocktail designs that consider individual patient conditions and infection site characteristics is desirable. Phage cocktail therapy is expected to be further developed as a promising option against antimicrobial resistance.
